# BLAP: lesion-aware adaptive multi-scale visual prompt tuning for few-shot crop disease diagnosis

**DOI:** 10.3389/fpls.2026.1901116

**Published:** 2026-08-17

**Authors:** Jiayi Xiong, Libin Li, Yiqing Wang, Dexi Chen, Lei Liu

**Affiliations:** 1 College of Agronomy, Sichuan Agricultural University, Chengdu, China; 2 College of Resources and Civil Engineering, Northeastern University, Shenyang, China; 3 Institute of Plant Protection, Sichuan Academy of Agricultural Sciences, Chengdu, China

**Keywords:** BLAP, crop disease diagnosis, few-shot learning, vision-language model, visual prompt tuning

## Abstract

**Introduction:**

Applying general-purpose vision-language models (VLMs) to crop disease diagnosis presents three critical bottlenecks: reliance on large-scale annotated data, the high computational cost of full finetuning, and existing adaptation methods designed mainly for discriminative classification without sufficient visual-linguistic interaction for generative diagnosis.

**Methods:**

We propose BLAP, an adaptive multi-scale visual prompt fine-tuning framework built upon BLIP-2. BLAP introduces an adaptive visual prompt fusion module (APFM) with learnable prompt vectors and a gating mechanism, together with a multi-scale pyramid feature fusion module (PFM). All BLIP-2 backbone parameters are frozen, and only 0.11% of the model parameters are optimized.

**Results:**

On a few-shot dataset comprising 990 images from 11 crops and 33 disease categories, BLAP achieved 92.78% recognition accuracy, outperforming the BLIP-2+LoRA baseline by 21.67 percentage points. BLEU-4 and ROUGE-L scores reached 0.6507 and 0.7184, respectively, while inference latency increased by only 2.15%.

**Discussion:**

BLAP provides a lightweight solution that balances accuracy, efficiency, and interpretability for crop disease diagnosis in resource-constrained settings. The proposed dynamic prompt fusion and multiscale pyramid adaptation strategy may also be extended to parameter-efficient fine-tuning of visionlanguage models in other domain-specific applications.

## 1 Introduction

Timely and accurate diagnosis of pests and diseases is a core task in agricultural plant protection. According to statistics, yield losses caused by pests and diseases for five major crops (wheat, rice, maize, potato, and soybean) range from roughly 10% to 41% globally, with a weighted average of approximately 21.5% to 30.0% (Savary et al., 2019). To reduce losses and enable effective preventive measures, methods for early pest and disease prediction are urgently needed (Strange and Scott, 2005).

Deep learning models that integrate convolutional neural networks with computer vision have been developed for agricultural pest and disease identification and diagnosis. Hassan Ali et al. (2021) use a fine-tuned EfficientNet-B0 to achieve 99.78% accuracy on the PlantVillage dataset and 99.69% on the apple leaf disease subset. An optimized ResNet-50 model with data augmentation techniques such as rotation, flipping, and scaling attains 98.7% accuracy on PlantVillage, demonstrating particularly strong performance on diseases like tomato early blight and potato late blight (Mohanty et al., 2016). These results, however, depend on 54,306 carefully annotated images, which incurs high labeling costs. Kamilaris and Prenafeta-Boldú, (2018) point out that the heavy reliance on large, manually annotated datasets severely constrains the application of deep learning in agriculture. Deep learning methods generally rely on large-scale expert-annotated datasets. However, acquiring field images in real agricultural environments is challenging, while accurate annotation requires extensive effort from plant pathology experts, resulting in high labor and time costs. Consequently, few-shot learning has become an important research direction for crop disease recognition (Sun et al., 2024). Unlike controlled datasets such as PlantVillage, field images of staple crops including wheat and cotton are often affected by complex backgrounds, illumination variations, leaf occlusion, and different stages of disease progression. These factors substantially increase annotation difficulty, making the construction of large-scale, high-quality agricultural datasets prohibitively expensive (Porto et al., 2023). To address this, Yang et al. (2025) propose a multimodal-guided few-shot learning (MMFSL) framework that aligns visual features with class name embeddings via a bilinear metric function, achieving 86.97% accuracy under a 5-way 1-shot setting on PlantVillage. Li et al. (2025) develop a few-shot crop disease recognition method based on a sequentially weighted ensemble model with a model-agnostic meta-learning (MAML) strategy and validate its effectiveness on multi-class crop disease datasets. Jiang et al. (2025) introduce PlantCaFo, which builds on a simplified CaFo architecture and integrates pretrained knowledge from multiple foundation models such as CLIP and DINO; using only four images per class sampled from PlantVillage for training, it obtains 82.63% accuracy. These studies, however, remain confined to discriminative tasks and can only output closed-set class labels. They are unable to provide fine-grained symptom descriptions or management recommendations, which limits their practical value in production.

Leveraging the combination of vision-language models (VLMs) and supervised learning (Zhang et al., 2024), AI systems achieve multimodal reasoning and discrimination capabilities. AgEval (Arshad et al., 2024) evaluates the performance of state-of-the-art VLMs (GPT-4V, Gemini, Claude, LLaVA) on 12 plant stress phenotyping tasks and finds that merely eight contextual examples substantially boost the F1 score of these models. AgEval operates as a recognition model based on black-box APIs without any parameter updates; its coefficient of variation (CV) in recognition accuracy across different disease categories ranges from 26.02% to 58.03%, indicating pronounced performance inconsistency of general-purpose VLMs across disease types. This underscores the urgent need for parameter optimization of vision-language models.

Full fine-tuning is a classical paradigm for transferring knowledge from large models and has achieved remarkable success on general vision tasks, yet its feasibility in agricultural research remains limited. Liu et al. (2025) explicitly state that full fine-tuning demands massive data and computational resources to adapt all parameters effectively, whereas agricultural scenarios involve diverse crop types, heterogeneous data modalities, and high sample collection costs, making this requirement difficult to satisfy.

Parameter-efficient fine-tuning (PEFT) techniques, such as LoRA (Low-Rank Adaptation), Adapter, and Prefix-Tuning, have been successfully applied to general vision-language tasks with notable effectiveness. For instance, Zahweh et al. (2023) demonstrate in a winter wheat segmentation task that full fine-tuning requires large-scale datasets, whereas PEFT achieves comparable performance using only 0.7% of the trainable parameters. Representative PEFT methods include Adapter, Prefix-Tuning, and LoRA, which adapt pretrained models by introducing lightweight trainable modules, learnable prompts, or low-rank weight updates while keeping most backbone parameters frozen (Houlsby et al., 2019; Li and Liang, 2021; Chen et al., 2022; Hu et al., 2022; Jia et al., 2022).

Recent PEFT approaches for vision-language models can generally be categorized into three paradigms: adapter-based methods, low-rank adaptation methods, and prompt learning methods. Adapter-based approaches improve downstream task adaptation by introducing lightweight trainable modules, whereas LoRA optimizes low-rank weight updates to reduce computational cost. Prompt learning methods instead adapt pretrained models by learning task-specific prompt representations without modifying backbone parameters. Existing PEFT methods mainly improve adaptation efficiency through lightweight parameter optimization, but they generally employ fixed prompt representations and overlook the multi-scale visual characteristics and dynamic cross-modal interaction required for generative crop disease diagnosis.

Recent studies have applied PEFT to agricultural disease diagnosis. Liu et al. (2025) proposed Dual-(PAL)G by jointly integrating prompt, adapter, and LoRA modules. Huang et al. (2026) improved LoRA through dynamic rank allocation to further reduce parameter redundancy. Kaur et al. (2025) combined lightweight vision-language adaptation with GroundingDINO and SAM-2 for tomato disease diagnosis. These studies remain primarily focused on elementary applications of PEFT to disease classification. They cannot provide corresponding symptom descriptions or feasible management recommendations for agricultural practitioners. Moreover, they lack a jointly designed adaptation strategy for multimodal feature fusion layers and prompt interaction layers, making them inadequate for the fine-grained, multi-scale, and strongly interactive demands of agricultural disease diagnosis.

To overcome these limitations, this work proposes BLAP, an adaptive multi-scale visual prompt tuning framework built upon BLIP-2. By introducing a pyramid feature fusion module and an adaptive prompt fusion module between the Q-Former and the language model, BLAP enables efficient disease recognition, symptom description, and treatment recommendation under few-shot settings.

Unlike existing PEFT frameworks that mainly focus on lightweight parameter optimization for discriminative visual tasks, BLAP explicitly models multi-scale pathological representations and dynamically injects content-aware visual prompts into the language model, enabling unified disease recognition, symptom description, and treatment recommendation within a single generative framework. Specifically, the proposed pyramid feature fusion module first extracts multi-scale pathological representations from leaf images, while the adaptive visual prompt fusion module transforms these representations into content-aware visual prompts that are dynamically injected into the language model. Consequently, the prompts are no longer static learnable parameters but adaptive semantic representations conditioned on disease characteristics. More importantly, these prompts not only enhance visual representation learning but also directly guide language generation, enabling unified disease recognition, symptom description, and treatment recommendation within a single vision-language model. This fundamentally distinguishes BLAP from existing visual prompt tuning frameworks designed primarily for discriminative classification tasks.

## 2 Materials and methods

### 2.1 Dataset description

The dataset constructed in this study consists of 990 images covering 11 crop species, including apple, cashew, cassava, chili, cotton, cucumber, grape, peanut, potato, soybean, and tomato, with 33 disease or health categories. All images are publicly available (**Table 1**). The images are collected from diverse settings, including laboratory and field environments (Approximately 45% of them were captured in field environments), to enhance sample representativeness and model robustness.

**Table 1:** The number of collected diseases or healthy image data for each crop.

Crop	Disease	No. of images	Collection conditions	Data source
Apple	Apple scab	30	Lab	https://data.mendeley.com/datasets/tywbtsjrjv/1
	Cedar apple rust	30	Lab	https://data.mendeley.com/datasets/tywbtsjrjv/1
	Healthy	30	Lab	https://data.mendeley.com/datasets/tywbtsjrjv/1
Cashew	Healthy	30	Lab	https://data.mendeley.com/datasets/8fr7grr73p/1
	Leaf miner	30	Lab	https://data.mendeley.com/datasets/8fr7grr73p/1
	Red rust	30	Lab	https://data.mendeley.com/datasets/8fr7grr73p/1
Cassava	Brown spot	30	Field	https://data.mendeley.com/datasets/8fr7grr73p/1
	Healthy	30	Lab	https://data.mendeley.com/datasets/8fr7grr73p/1
	Mosaic	30	Field	https://data.mendeley.com/datasets/8fr7grr73p/1
Chilli	Healthy	30	Field	https://data.mendeley.com/datasets/8fr7grr73p/1
	Nutrition Deficiency	30	Field	https://data.mendeley.com/datasets/8fr7grr73p/1
	White spot	30	Field	https://data.mendeley.com/datasets/8fr7grr73p/1
Cotton	Bacterial Blight	30	Lab	https://data.mendeley.com/datasets/8fr7grr73p/1
	Curl Virus	30	Field	https://data.mendeley.com/datasets/8fr7grr73p/1
	Healthy	30	Mix	https://data.mendeley.com/datasets/8fr7grr73p/1
Cucumber	Healthy	30	Field	https://data.mendeley.com/datasets/y6d3z6f8z9/1
	Bacterial Wilt	30	Field	https://data.mendeley.com/datasets/y6d3z6f8z9/1
	Anthracnose	30	Field	https://data.mendeley.com/datasets/y6d3z6f8z9/1
Grape	Black rot	30	Lab	https://data.mendeley.com/datasets/tywbtsjrjv/1
	Esca	30	Lab	https://data.mendeley.com/datasets/tywbtsjrjv/1
	Healthy	30	Lab	https://data.mendeley.com/datasets/tywbtsjrjv/1
Groundnut	Healthy	30	Field	https://data.mendeley.com/datasets/8fr7grr73p/1
	Late leaf spot	30	Field	https://data.mendeley.com/datasets/8fr7grr73p/1
	Nutrition deficiency	30	Field	https://data.mendeley.com/datasets/8fr7grr73p/1
Potato	Early blight	30	Lab	https://data.mendeley.com/datasets/tywbtsjrjv/1
	Healthy	30	Lab	https://data.mendeley.com/datasets/tywbtsjrjv/1
	Late blight	30	Lab	https://data.mendeley.com/datasets/tywbtsjrjv/1
Soyabean	Frog Leaf Eye	30	Field	https://data.mendeley.com/datasets/hkbgh5s3b7/1
	Healthy	30	Field	https://data.mendeley.com/datasets/hkbgh5s3b7/1
	Mosaic	30	Field	https://data.mendeley.com/datasets/hkbgh5s3b7/1
Tomato	Healthy	30	Lab	https://data.mendeley.com/datasets/tywbtsjrjv/1
	Leaf Mold	30	Lab	https://data.mendeley.com/datasets/tywbtsjrjv/1
	Septoria leaf spot	30	Lab	https://data.mendeley.com/datasets/tywbtsjrjv/1

**Figure 1:**
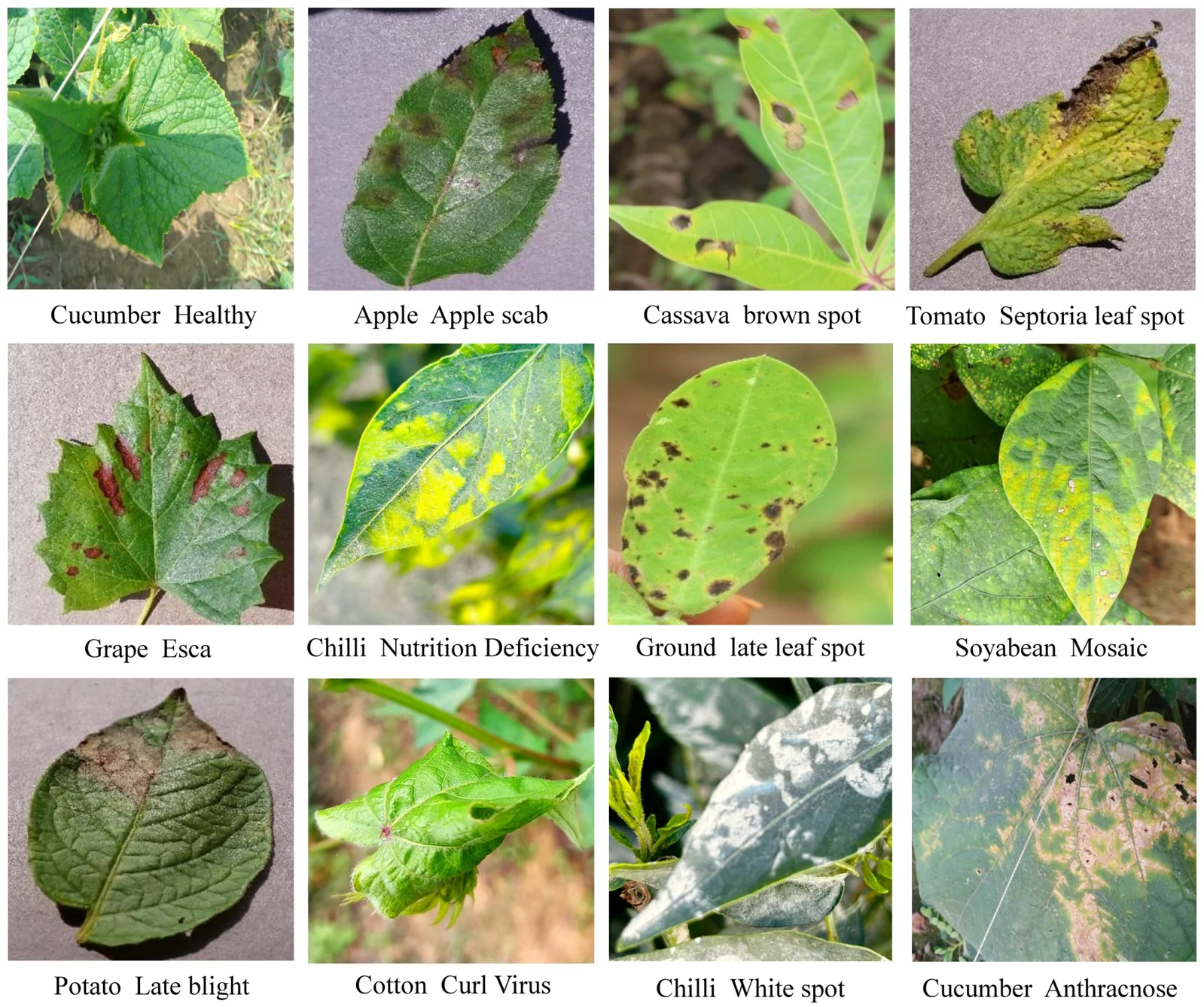
Illustration of partial data samples.

These images originate from several well-known datasets, including those produced by Daffodil International University, Jahangirnagar University, Malaviya National Institute of Technology Jaipur, Bikaner Technical University, as well as the publicly available datasets MH-SoyaHealthVision and PlantVillage. The dataset is compiled from multiple public sources, which introduces the risk of visually similar duplicate images across different sources. If the training and test sets were partitioned directly without addressing this issue, data leakage would occur and consequently inflate the reported model performance. To prevent this, we perform a rigorous deduplication audit prior to data partitioning. We first compute the MD5 and SHA-256 hash for each image to detect exact duplicate files. For near duplicates, we compute the perceptual hashes, namely pHash and dHash, and calculate the Hamming distance between every pair of images. Any pair with a distance below a predetermined threshold is marked as a near-duplicate candidate, and all such candidates are grouped into the same duplicate cluster. In the subsequent partitioning, we enforce that all images belonging to the same cluster are assigned entirely to either the training set or the test set, never straddling both. After partitioning, we conduct a secondary verification to check for any cross-split leakage and verify that no such leakage is present. With the deduplicated data, we adopt stratified random sampling to split the dataset into training and test subsets at an 8:2 ratio. The final dataset comprises 990 images in total, with each disease category providing 24 training samples and 6 test samples. **Figure 1** displays twelve representative images from the training subset (A. P. and Gopal; Jain; Shinde et al.; Sultana et al.; Hughes and Salathé, 2015; Geetharamani and Pandian, 2019).

To overcome the tendency of few-shot training to cause overfitting and poor generalization, this study adopts a real-time data augmentation strategy based on the torchvision.transforms library (Paszke et al., 2019). Augmentation operations are applied only to the training set, while the test set retains the original data distribution to ensure reliable and realistic evaluation. This strategy effectively increases the diversity of training samples and the model robustness under complex real-world conditions, alleviating overfitting in low-data regimes. The augmentation operations include random resized crop, random horizontal flip, random rotation, and random adjustments of brightness, contrast, saturation, and hue. In few-shot scenarios, our proposed real-time augmentation method shows significant advantages over offline augmentation. Instead of the traditional pre-generate-and-store paradigm, this method adopts an on-the-fly dynamic strategy: a random combination of transformations with varying random intensities is applied in each training epoch. This design ensures that the augmented samples fed into the model in every epoch are globally unique, producing a vast amount of training data with diverse visual appearances yet semantically invariant features, which suffices to mitigate overfitting caused by small training sets.

### 2.2 Theoretical preliminaries

#### 2.2.1 BLIP-2 vision-language pre-trained model

BLIP-2 is a mainstream lightweight vision-language model (VLM) (Li et al., 2023) whose core innovation lies in bridging a fully frozen visual encoder and a large language model (LLM) through a lightweight Querying Transformer (Q-Former), thereby circumventing the prohibitive computational cost and overfitting risk of full fine-tuning (Dai et al., 2023). The architecture of BLIP-2 consists of two core stages:

The first stage is vision-language representation learning. A frozen EVA-CLIP visual encoder serves as the feature extractor (Sun et al., 2023). An input image $I\in {\mathbb{R}}^{H\times W\times 3}$ passes through the encoder and produces visual features $V\in {\mathbb{R}}^{L_{v}\times d_{v}}$, where $L_{v}$ denotes the sequence length of the visual features and $d_{v}$ denotes the feature dimension. The Q-Former uses a set of learnable query vectors $Q\in {\mathbb{R}}^{N_{q}\times d_{q}}$as an intermediary and interacts with the visual features through cross-attention, yielding visual representations $V_{ql}\in {\mathbb{R}}^{N_{q}\times d_{q}}$that are aligned with textual semantics. The core attention computation is given by Equation 1:

(1) $$\text{Attention}(Q,K,V)=\text{Softmax}(\frac{QK^{T}}{\sqrt{d_{k}}})V$$

where Q denotes the query vectors, K and V denote the key and value matrices mapped from the visual features, and $d_{k}$is the dimension of the key vectors. The second stage is vision-to-language generation learning. The visual representations output by the Q-Former are directly prepended to the text sequence and fed into a frozen LLM (OPT-2.7B is adopted in this work), guiding the LLM to perform text generation conditioned on visual input.

In its original form, BLIP-2 relies on a simple feature concatenation scheme for image-text interaction. It is not adapted to the multi-scale visual characteristics of agricultural diseases or to the requirements of domain-specific generative tasks. It can cover neither fine-grained plant lesion features nor coarse-grained leaf necrosis characteristics simultaneously, and the visual modality provides only weak guidance for language generation. These limitations constitute the core optimization directions of this work.

#### 2.2.2 Low-rank adaptation (LoRA)

LoRA is a mainstream parameter-efficient fine-tuning (PEFT) method (Mangrulkar et al., 2022) whose core strategy is to freeze all backbone weights of the pretrained model and insert only trainable low-rank decomposition matrices into the query (Q) and value (V) projection matrices of Transformer self-attention layers. This design substantially reduces the number of trainable parameters while mitigating the overfitting risk caused by small training sets.

For a pretrained weight matrix $W_{0}\in {\mathbb{R}}^{d_{in}\times d_{out}}$, LoRA decomposes its update into two low-rank matrices $B\in {\mathbb{R}}^{d_{in}\times r}$ and $A\in {\mathbb{R}}^{r\times d_{out}}$, where the rank $r\ll \min(d_{in},d_{out})$, The forward pass output is given by Equation 2:

(2) $$h=xW_{0}+xBA$$

where xdenotes the layer input and h denotes the layer output. At inference time, the low-rank update BA can be merged into the original weights $W_{0}$.

### 2.3 Overall architecture of the proposed framework

**Figure 2:**
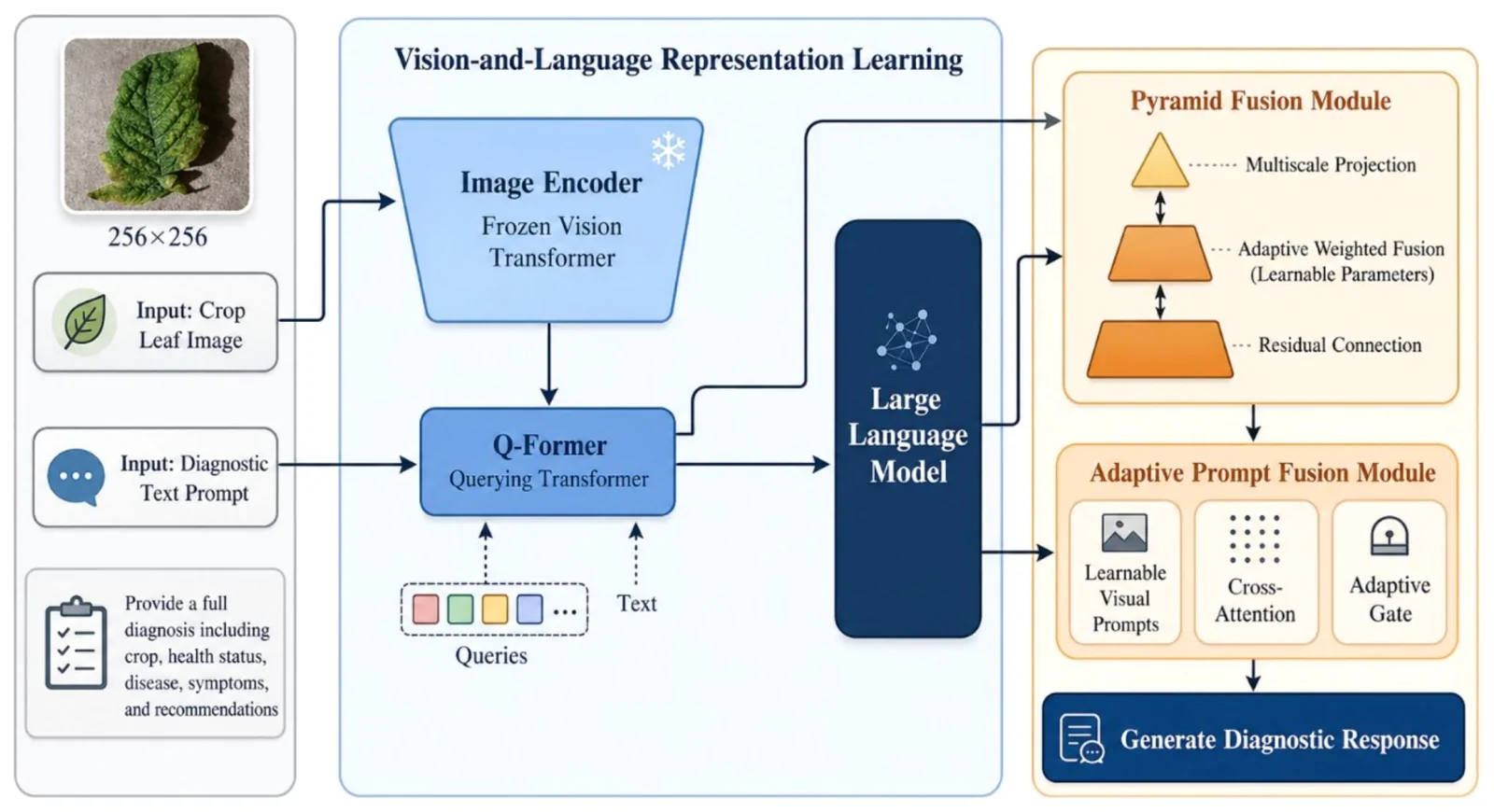
Overall architecture of the proposed multi-dimensional efficient fine-tuning framework for few-shot crop disease diagnosis.

General-purpose VLMs suffer from several critical shortcomings in few-shot crop disease diagnosis: inadequate capacity for capturing multi-scale disease features, weak guidance of visual information on text generation, and prohibitively high costs of full fine-tuning. To address these issues, this work proposes a multi-dimensional efficient fine-tuning framework built upon BLIP-2, with the overall architecture illustrated in **Figure 2**.

The framework adopts BLIP-2 with OPT-2.7B as the backbone. While keeping the visual encoder and the main weights of the large language model frozen, it introduces a lightweight parameter-efficient adaptation mechanism to enable capability transfer for few-shot crop disease diagnosis. Specifically, the model first employs the frozen Vision Transformer to extract visual representations of crop leaf images and aligns these visual features with diagnostic text prompts through the Q-Former, producing cross-modal representations that the LLM can interpret. Building on this, the framework designs a multi-scale pyramid feature fusion module to capture multi-scale visual cues such as fine-grained lesion textures, local spreading regions, and global leaf necrosis states, thereby enhancing the expressive capacity of visual features for complex disease phenotypes. In parallel, an adaptive visual prompt fusion module, through learnable visual prompts, cross-attention interaction, and an adaptive gating mechanism, dynamically modulates the guidance intensity of visual information on the diagnostic text generation process, enabling the model to produce structured diagnostic outputs covering crop type, health status, disease symptoms, and management recommendations. Both fusion modules are non-intrusively embedded into the forward pass of BLIP-2 via forward hook mechanisms. During training, only the LoRA low-rank matrices and the trainable parameters within the two fusion modules are updated.

This framework achieves non-intrusive integration via two forward hooks. The first hook is registered at the output of Q-Former to capture multi-scale visual features. The second hook is attached to the end of the OPT-2.7B decoder, after all twelve decoder layers have completed computation. At this point, the fusion module combining PFM and APFM operates on the final hidden states produced by the decoder, and the resulting representations are then fed into the LM Head for text generation. The fusion module is not inserted between individual decoder layers but is executed only once at the decoder terminus, thereby preserving the integrity of the main model’s forward computation flow.

### 2.4 Multi-scale pyramid feature fusion module

#### 2.4.1 Module design motivation

**Figure 3:**
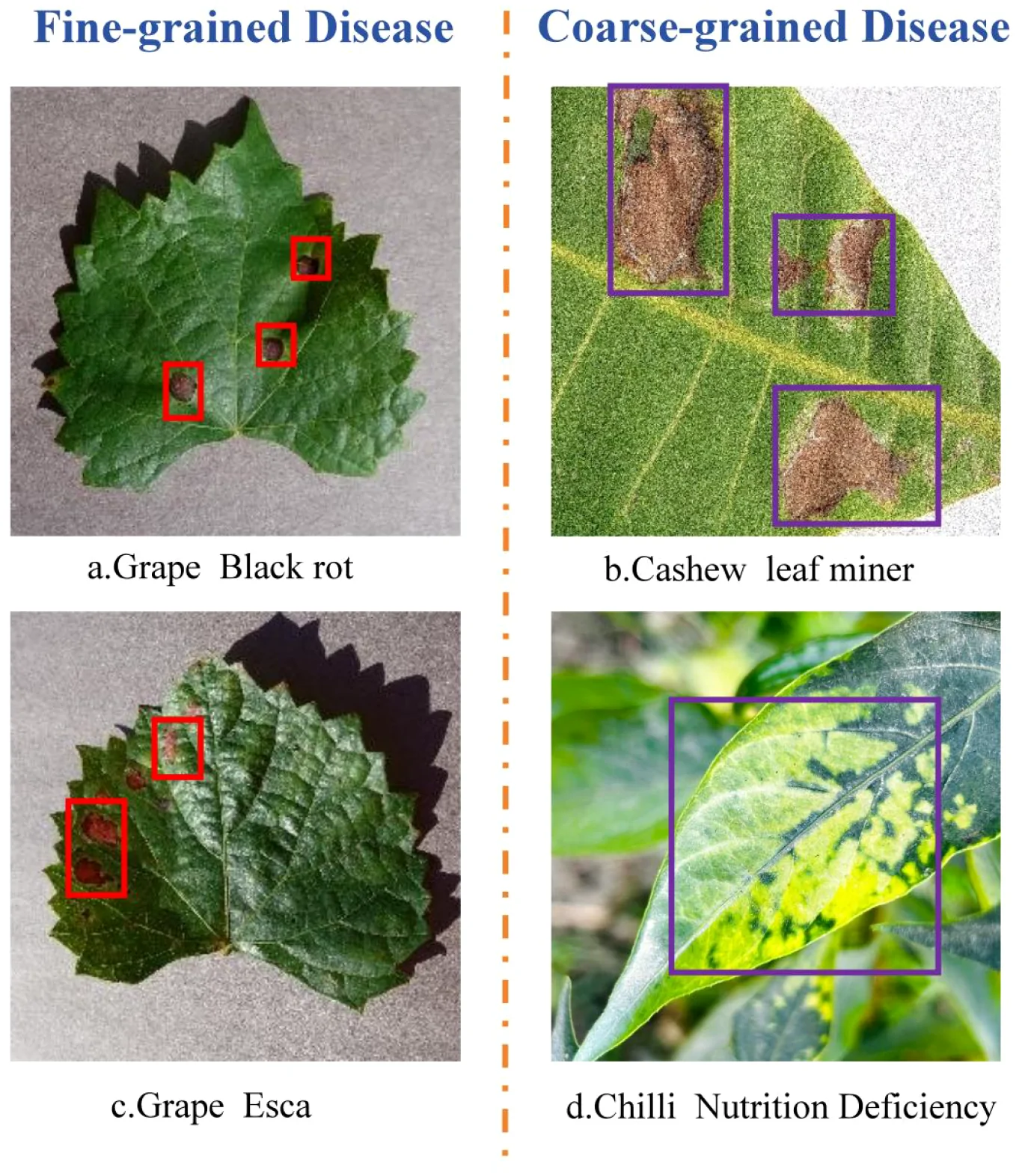
Examples of fine-grained and coarse-grained crop disease symptoms: **(a)** grape black rot, **(b)** cashew leaf miner, **(c)** grape Esca, and **(d)** chilli nutrition deficiency.

Visual features of crop diseases naturally exhibit a multi-scale nature. Fine-grained features, such as pinpoint lesions from scab or bacterial spot, occupy only 1% to 5% of the leaf area. Coarse-grained features, such as extensive necrosis and whole-leaf yellowing from late blight or leaf blight, cover more than 50% of the leaf area. **Figure 3** shows examples of four disease categories across three crops: (a) grape black rot, (b) cashew leaf miner damage, (c) grape Esca disease, and (d) chilli nutrition deficiency. These examples reveal that the fine-grained visual differences among diseases are substantial and clearly multi-scale. Moreover, different diseases affecting the same crop sometimes present highly similar symptoms. For instance, in **Figure 3**, grape black rot (a) and grape Esca disease (c) both manifest as irregular brown necrotic spots on leaves, making them visually similar. Such visual confusion between different diseases on the same crop greatly increases the difficulty of automated recognition. Therefore, in the context of agricultural disease diagnosis, a model must possess multi-scale feature extraction capabilities to effectively distinguish such highly similar fine-grained categories.

**Figure 4:**
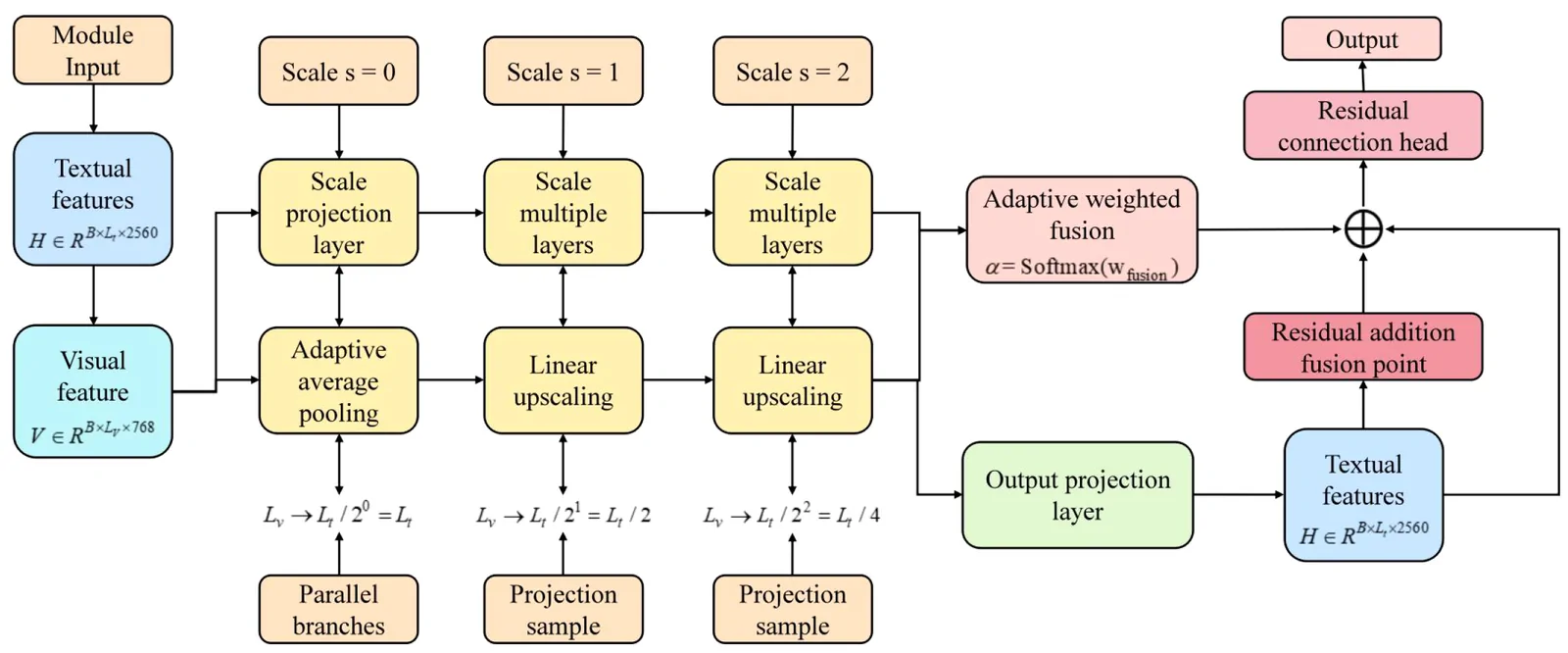
Structure of the proposed multi-scale pyramid feature fusion module.

The Q-Former in the original BLIP-2, however, outputs only single-scale visual features and can cover neither fine-grained nor coarse-grained disease information simultaneously, which easily leads to missed detection of critical lesions and disease misclassification. Meanwhile, the simple feature concatenation scheme in the original design fails to achieve deep fusion of visual and textual features, resulting in insufficient guidance of visual information on language generation. To address these limitations, this work designs a multi-scale pyramid feature fusion module that enables multi-scale disease feature extraction and deep vision-language fusion. The module structure is illustrated in **Figure 4**.

#### 2.4.2 Module implementation principle

The module takes as input the visual features $V_{ql}\in {\mathbb{R}}^{B\times L_{v}\times 768}$output by the Q-Former and the textual features $H_{text}\in {\mathbb{R}}^{B\times L_{t}\times 2560}$output by the LLM decoder, where B denotes the batch size and $L_{t}$denotes the text sequence length. It produces fused textual features $H_{pyramid}$ enriched with multi-scale visual information through the following four steps.

Step 1: Feature space alignment.

Since the dimensions of visual and textual features are mismatched, direct fusion is infeasible. Three parallel linear projection layers map the 768-dimensional visual features to 2560 dimensions, matching the textual feature space, as shown in Equation 3:

(3) $$V_{proj}^{\left(s\right)}={\text{Linear}}^{\left(s\right)}(V_{ql}),\;s=0,1,2$$

where $V_{proj}^{\left(s\right)}$denotes the visual features from the slinear projection and ${\text{Linear}}^{\left(s\right)}$denotes the corresponding projection layer. Layer normalization, ReLU activation, and dropout are applied after each projection to stabilize the feature distribution and mitigate overfitting.

Step 2: Multi-scale feature extraction.

Adaptive average pooling extracts features at different granularities from the three projected feature branches. Linear interpolation then upsamples all pooled features to the same sequence length L as the textual features, achieving alignment along the sequence dimension, as defined in Equations 4 and 5:

(4) $$V_{pool}^{\left(s\right)}={\text{AvgPool}}_{k=2^{s}}(V_{proj}^{\left(s\right)})$$

(5) $$V_{scale}^{\left(s\right)}=\text{Upsample}(V_{pool}^{\left(s\right)},size=L_{t})$$

where s=0 preserves the original sequence length to capture fine-grained temporal correlations; s=1compresses the sequence to half its original length to capture mid-range dependencies; and s=2compresses the sequence to one quarter to capture long-range relationships and holistic variation patterns. The three scales directly correspond to the natural hierarchical distribution of visual features for crop diseases. The downsampling ratios of 1/2 and 1/4 relative to the original resolution follow an equal multiplicative progression with a factor of two between consecutive scales, such that the three scales uniformly cover the semantic span required for disease discrimination across feature granularities while avoiding excessive redundancy. Within our framework, this three-scale configuration is identified as the optimal choice.

Step 3: Adaptive weighted fusion.

To enable adaptive selection over multiple feature scales, learnable fusion weights are introduced and normalized via a softmax function. The model automatically learns the multi-scale weighting scheme for different disease types during training, as formalized in Equations 6 and 7:

(6) $$\alpha^{\left(s\right)}=\text{Softmax}(w_{fusion}^{\left(s\right)}),\;s=0,1,2$$

(7) $$V_{fused}=\sum_{s=0}^{2}\;\alpha^{\left(s\right)}\cdot V_{scale}^{\left(s\right)}$$

where $w_{fusion}^{\left(s\right)}$denotes the learnable weight coefficients, $\alpha^{\left(s\right)}$ denotes the normalized fusion weights, and $V_{fused}$denotes the weighted multi-scale visual features. For instance, when identifying apple scab, the fine-grained scale weight strengthens automatically; when identifying potato late blight, the coarse-grained scale weight strengthens, achieving feature self-adaptation.

Step 4: Residual fusion output.

To avoid compromising the text semantic generation capability of the LLM, a residual connection fuses the multi-scale visual features with the original textual features, as given in Equation 8:

(8) $$H_{pyramid}={\text{Linear}}_{out}(V_{fused})+H_{text}$$

where ${\text{Linear}}_{out}$ is an output projection layer that refines the distribution of the fused features, and $H_{pyramid}$ denotes the final output of the pyramid fusion module.

### 2.5 Adaptive visual prompt fusion module

#### 2.5.1 Module design motivation

The original BLIP-2 achieves image-text interaction solely by prepending visual features to the text sequence, which introduces two fundamental limitations. First, the simple concatenation of visual and textual features precludes deep cross-modal interaction, making the language model prone to overlooking critical visual details during generation. Second, the degree to which visual information is injected remains fixed and cannot be dynamically adjusted according to the semantics of the content being generated; for instance, generating symptom descriptions demands stronger visual guidance, whereas generating management recommendations relies more on general linguistic knowledge.

**Figure 5:**
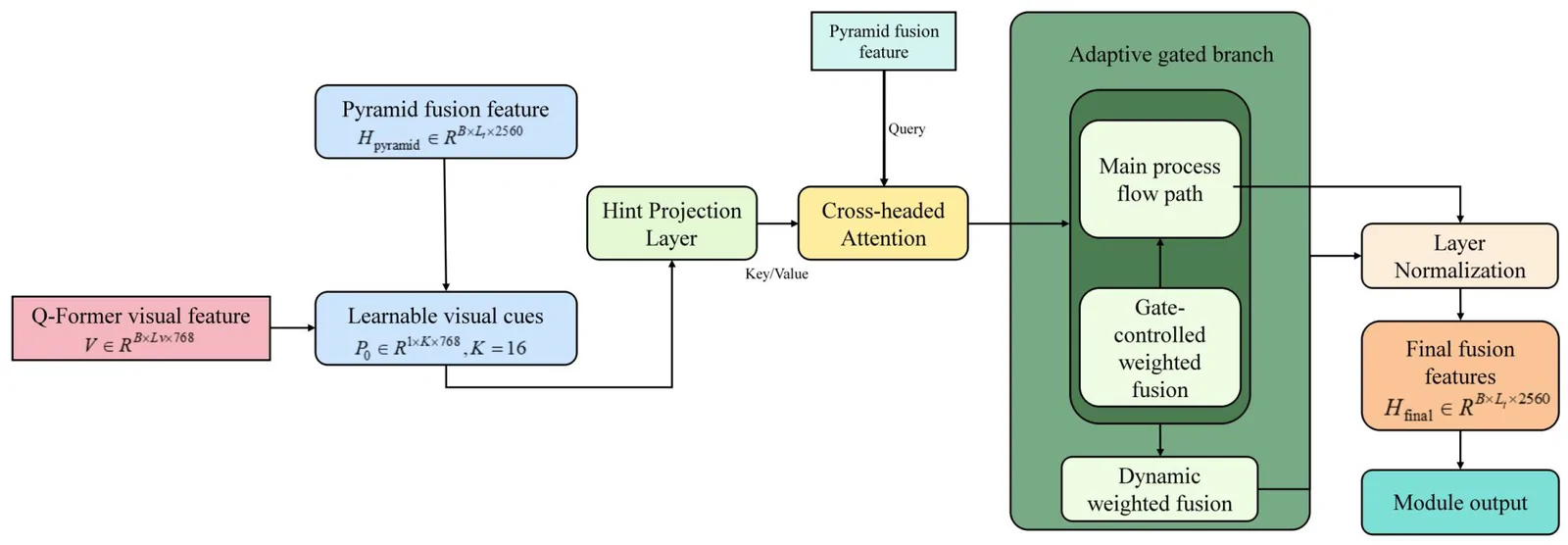
Structure of the proposed adaptive visual prompt fusion module.

To address these limitations, this work designs an adaptive visual prompt fusion module that enables flexible guidance of visual information on language generation through learnable visual prompt vectors and an adaptive gating mechanism. The module structure is illustrated in **Figure 5**.

#### 2.5.2 Module implementation principle

The module takes as input the textual features from the pyramid fusion module and the visual features from the Q-Former, and outputs fused textual features. The core implementation consists of five sequential steps.

First, learnable visual prompt vectors are initialized as a two-dimensional matrix of size 1 × K × 768, where K = 16 denotes the number of prompt vectors. These prompt vectors are globally shared learnable parameters across all crop and disease categories, rather than being learned independently for each disease class. This design enables the prompt vectors to capture generic visual concepts of crop diseases, such as lesions, yellowing, and mildew layers, which are co-occurring visual patterns across categories, instead of memorizing category-specific features. Such a strategy mitigates overfitting and improves generalization under few-shot conditions. During forward propagation, the prompt vectors are expanded to match the batch dimension, ensuring that every sample within the batch shares the same set of prompt vectors. To enable attentive interaction between the visual prompts and text features, a linear projection layer maps the prompt vectors from 768 dimensions to 2560 dimensions, aligning them with the text feature space as shown in Equation 9:

(9) $$P_{proj}={\text{Linear}}_{prompt}(P_{0})$$

where ${\text{Linear}}_{prompt}$denotes the prompt projection layer, followed by layer normalization, ReLU activation, and dropout to stabilize the prompt feature distribution.

Second, a multi-head cross-attention module enables deep interaction between the textual features and the visual prompts. The textual features after pyramid fusion serve as the query, while the projected visual prompts serve as the key and value, allowing the textual features to actively attend to visual prompt information relevant to the current generation content, as specified in Equations 10 and 11:

(10) $$A=\text{MultiHeadAttention}(Q=H_{pyramid},K=P_{proj},V=P_{proj})$$

(11) $$\text{MultiHeadAttention}=\text{Concat}(head_{1},\ldots ,head_{h})W_{o}$$

where A denotes the attention output, *h* = 8 is the number of attention heads, and $W_{o}$is the output projection matrix. Through this attention mechanism, when generating content such as ``black spots on the leaf,’’ the textual features automatically focus on the prompt vectors corresponding to relevant disease concepts, substantially improving the efficiency of visual information guidance.

Third, to achieve dynamic adjustment of the degree of visual information injection, a learnable adaptive gating mechanism is designed. It outputs a gating value between 0 and 1 that dynamically determines the proportion of visual information at the current generation position. The core computations are given in Equations 12–14:

(12) $$C=\text{Concat}(H_{pyramid},A)$$

(13) $$g=\text{Sigmoid}({\text{MLP}}_{gate}(C))$$

(14) $$H_{gate}=g\cdot A+(1-g)\cdot H_{pyramid}$$

where C denotes the concatenation of the original textual features and the attention features, ${\text{MLP}}_{gate}$denotes the gating MLP, g denotes the gating value, and $H_{gate}$ denotes the gated fusion output. When g approaches 1, the model relies predominantly on visual prompt features, suiting generation scenarios that require visual detail, such as disease name and symptom description. When g approaches 0, the model relies predominantly on textual features, suiting scenarios that draw on general linguistic knowledge, such as management recommendations. The gating network adopts a two-layer fully connected architecture. Its input is the concatenated vector [h;a] of the original text features and the cross-attention output features. The gating values are generated token-wise along the sequence dimension, where each token position yields an independent scalar weight g∈ [0,1]. This enables fine-grained control over the intensity of visual information injection at different generation positions. It is worth noting that the input features to the gating module have already undergone preliminary cross-modal semantic association; the responsibility of the gating module is solely to produce a scalar control coefficient according to the current generation context, rather than to perform deeper feature interaction tasks.

Finally, layer normalization is applied to the gated fusion features to stabilize the feature distribution and prevent gradient vanishing or explosion during training, as given in Equation 15:

(15) $$H_{final}=\text{LayerNorm}(H_{gate})$$

where $H_{final}$ denotes the final output of the adaptive prompt fusion module, which replaces the original output of the LLM decoder and is fed into the subsequent LM head layer for the final generation task.

### 2.6 Training strategy and implementation details

#### 2.6.1 Loss function

A standard autoregressive language modeling loss, i.e., cross-entropy loss, serves as the sole optimization objective for model training. Given an input text sequence $Y=[y_{1},y_{2},\ldots ,y_{n}]$, the optimization objective is to minimize the negative log-likelihood of predicting the next token, as formulated in Equation 16:

(16) $$L=-\frac{1}{n}\sum_{i=1}^{n}\log p(y_{i}|y_{<i},I;\theta )$$

where I denotes the input crop leaf image and θ denotes the trainable parameters of the model, specifically the LoRA matrices and the parameters of the pyramid fusion module and the adaptive prompt fusion module. All parameters of the proposed modules are learned end-to-end through this single loss function without any auxiliary losses, ensuring a streamlined training procedure and stable convergence in few-shot settings.

#### 2.6.2 Training strategy

During training, all parameters of the BLIP-2 visual encoder, the Q-Former backbone, and the LLM decoder backbone remain strictly frozen. Trainable parameters are updated along only three paths. The LoRA path consists of low-rank decomposition matrices of rank 8 inserted into the Q and V projection matrices of the OPT-2.7B self-attention layers. The pyramid fusion module path includes the multi-scale linear projection layers, the learnable adaptive fusion weights, and the output projection layer, totaling roughly 5.9 million trainable parameters. The adaptive prompt fusion module path comprises the learnable visual prompt vectors, the prompt projection layer, the multi-head cross-attention layer, and the gating MLP, containing approximately 21 million trainable parameters. The combined trainable parameters amount to roughly 31 million, which constitutes merely 0.11% of the total parameters of the full BLIP-2-OPT-2.7B model.

The LoRA configuration is as follows: the rank is set to r=8, the scaling factor to α = 32, and the target modules are the query projection and value projection within the self-attention layers of the OPT-2.7B decoder, while the key and output projections remain frozen. It should be emphasized that all parameters of Q-Former are completely frozen and not subjected to LoRA, so as to preserve its cross-modal alignment capability as a vision-language bridge. A precise calculation yields 983,040 trainable parameters from LoRA, specifically 12 layers×2 projections×2×8×2560 = 983,040, which accounts for approximately 0.0035% of the total model parameters.

In addition, 4-bit quantization is applied to the LLM backbone weights to further reduce memory consumption, enabling training on a consumer-grade RTX 4060 8GB GPU. All experiments are implemented with PyTorch 2.7.1+cu118 and Hugging Face Transformers 4.57.6. The AdamW optimizer is used with a weight decay of 0.01 and an initial learning rate of 2e-4. A linear warmup covers 10% of the total training steps, followed by a cosine decay schedule. The per-GPU batch size is set to 1 with gradient accumulation over 8 steps, yielding an effective batch size of 8, gradient clipping threshold is set to 1.0. Training runs for 20 epochs and performance is evaluated at the end of each epoch. Double quantization with 4-bit NF4 is adopted to minimize memory footprint. During inference, the low-rank LoRA matrices are merged into the native LLM weights, and the parameters of the two fusion modules are integrated into the forward pass. This provides preliminary evidence for subsequent deployment on edge devices.

To prevent overfitting under few-shot conditions, multiple regularization strategies are employed throughout training. The low-rank constraint of LoRA with 8 structurally limits the parameter space. A dropout layer with a rate of 0.1 is applied after each linear layer within the fusion modules. The optimizer is AdamW with a weight decay of 0.01, and the gradient clipping threshold is set to 1.0. The learning rate schedule combines a warmup over 10% of the total steps with cosine annealing. In addition, the real-time data augmentation strategy described in Section 2.1 also serves a regularizing effect, further enhancing model generalization by increasing the diversity of training samples.

### 2.7 Experimental setting

This study adopts a pretrained BLIP-2 model with the OPT-2.7B language decoder as the backbone. The detailed hardware configuration, software environment, and training hyperparameters for all experiments are listed in **Table 2**.

**Table 2:** Detailed configuration of the experimental environment and training hyperparameters.

Category	Parameter	Value
Hardware Configuration	Hardware Device	NVIDIA GeForce RTX 4060 Laptop GPU
	VRAM Size	8GB
	Quantization Configuration	4-bit Quantization (BitsAndBytesConfig)
	Quantization Type	nf4
	Computation Data Type	float16
Software Environment	Operating System	Microsoft Windows 11
	Python	3.9
	CUDA	11.8
	Torch	2.7.1+cu118
	torchvision	0.22.1+cu118
	Transformers	4.57.6
	Pillow	11.3.0
	Requests	2.32.5
Training Hyperparameters	Optimizer	AdamW
	Learning Rate	2e-4
	Weight Decay	0.01
	Learning Rate Scheduler	Cosine Annealing with 10% Warmup Ratio
	Training Epochs	20
	Single-GPU Batch Size	1
	Gradient Accumulation Steps	8
	Effective Batch Size	8
	Trainable Parameters	31M (0.11% of full model)
	Training VRAM Usage	~7.2 GB (with 4-bit quantization)
	Training Time (20 epochs)	~6.5 hours
Image Augmentation Configuration	Gradient Clipping	1.0
	Random Resized Crop Scale	[0.7, 1.0]
	Random Horizontal Flip	p=0.5
	Random Rotation	[-30°, +30°]
	Color Jitter (Brightness)	± 0.3
	Color Jitter (Contrast)	± 0.3
	Color Jitter (Saturation)	± 0.3
	Color Jitter (Hue)	± 0.1

To ensure reproducibility and statistical reliability, all experiments are repeated with three independent random seeds, namely 42, 1234, and 2023. For each seed, the complete procedure includes dataset partitioning via stratified sampling, model initialization, and training, followed by evaluation. The reported performance metrics are presented as the mean and standard deviation computed over the three independent runs. All compared models are evaluated under the same set of random seeds to guarantee fair comparison.

To verify whether the performance improvement of our method over the baseline model (BLIP-2+LoRA) is statistically significant, we conduct two-tailed paired t-tests on the paired results of five evaluation metrics across three independent repeated experiments, with the significance level set at α=0.05. All pairings are established on a one-to-one basis under identical random seeds, so as to effectively control the influence of random fluctuations on the conclusions.

### 2.8 Evaluation indicators

Five core metrics are selected to form a comprehensive evaluation framework for the multimodal few-shot crop disease diagnosis task. These metrics collectively capture the model’s disease identification performance, the interpretability of its diagnostic output, and its feasibility for deployment on edge devices, thereby reflecting the overall performance from the perspectives of accuracy, practical utility, and efficiency. The five evaluation metrics are defined in Equations 17–21.

(17) $$Acc_{both}=\frac{1}{N}\sum_{i=1}^{N}1({\hat{c}}_{i}=c_{i}\wedge {\hat{d}}_{i}=d_{i})$$

(18) $$\text{BLEU}-4=\text{BP}\cdot \exp(\frac{1}{4}\sum_{n=1}^{4}\log p_{n})$$

(19) $$\text{ROUGE-L}=\frac{2\cdot R_{lcs}\cdot P_{lcs}}{R_{lcs}+P_{lcs}}$$

(20) $$\text{SRR}=\frac{N_{matched}^{symptom}}{N_{total}^{symptom}}\times 100\%$$

(21) $$\text{AIL}=\frac{\sum_{i=1}^{N_{total}}t_{i}}{N_{total}}$$

Let *N* denote the total number of test samples, *ci* and *di* represent the true crop category and disease category of the *i-th* sample, and $\overset{\wedge }{ci}$ $\overset{\wedge }{di}$denote the crop category and disease category extracted from the model’s generated diagnostic text. The indicator function 1()returns 1 if both the crop and disease categories are correctly predicted, and 0 otherwise. BP denotes the brevity penalty that prevents inflated scores from overly short generations, and $p_{n}$ denotes the n-gram matching precision of the generated text against the reference. $R_{lcs}$ and $P_{lcs}$ denote the recall and precision computed from the longest common subsequence between the generated and reference texts. $N_{matched}^{symptom}$ and $N_{total}^{symptom}$ denote the number of correctly matched pathological symptom keywords across all disease categories and the total number of predefined symptom keywords, respectively. $t_{i}$ denotes the end-to-end inference latency of the i-th test sample in milliseconds, measured from leaf image input to the completion of diagnostic text output. Additionally, per-class accuracy is computed independently for each disease category.

Given that the generation task aims to produce structured diagnostic text, we adopt BLEU-4, ROUGE-L, and symptom recall to measure the similarity between generated content and ground truth answers as well as key information coverage. BLEU-4 is the BLEU score based on 4-grams, evaluating n-gram overlap between the generated text and the reference answer. We use a simplified implementation without NLTK dependency, computing the geometric mean with a length penalty. ROUGE-L is the ROUGE score based on LCS, measuring content fluency and information coverage. Symptom recall is computed for each disease category by extracting a set of symptom keywords from the ground truth answer in advance and calculating the proportion of these keywords present in the generated text, reflecting the model’s accuracy in symptom description. The efficiency metric is average inference latency, recorded as the milliseconds needed from input image to complete text generation for each test sample, averaged over all samples.

In addition to the five existing metrics, namely accuracy, BLEU-4, ROUGE-L, symptom recall, and average inference latency, we report the mean and standard deviation for each metric over multiple independent runs to enhance the statistical reliability of the results. For a given metric, suppose the values obtained from K independent runs are denoted as *m_k_*, k=1,2,…,K, with K = 3 in this work. The mean and standard deviation are then computed according to Equations 22 and 23, respectively.

(22) $$\text{mean}=\frac{1}{K}\sum_{k=1}^{K}m_{k}$$

(23) $$\text{std}=\sqrt{\frac{1}{K-1}\sum_{k=1}^{K}{\left(m_{k}-\text{mean}\right)}^{2}}$$

To verify whether the performance improvement of our method over the baseline model is statistically significant, we employ two-tailed paired t-tests on the evaluation metrics from the core comparative experiments, with the significance level set at α=0.05 For a given metric, let $d_{i}=x_{i}^{BLAP}-x_{i}^{baseline}(i=1,2,3)$ denote the performance difference between BLAP and the baseline in the i-th independent experiment. The test statistic and the associated mean difference and standard deviation are computed as shown in Equations 24–26:

(24) t=d−sdn

(25) $$\overset{-}{d}=\frac{1}{n}\sum_{i=1}^{n}d_{i}$$

(26) $$s_{d}=\sqrt{\frac{1}{n-1}\sum_{i=1}^{n}(d_{i}-{\overset{+}{d)}}^{2}}$$

Here n=3 denotes the number of independent repeated experiments, and the degrees of freedom are v=n−1 = 2. All pairings are established on a one-to-one basis under the same random seeds, so as to effectively control the influence of random fluctuations on the conclusions.

## 3 Results

### 3.1 Comparison experiment

We train and evaluate the proposed BLAP model under identical few-shot dataset settings, hardware environment, and training hyperparameters, and directly compare it with the baseline BLIP-2 model fine-tuned with LoRA (denoted as BLIP2+LoRA) as shown in **Table 3**.

**Table 3:** Quantitative performance comparison between the proposed BLAP model and the BLIP2+LoRA baseline.

Evaluation metrics	BLAP	BLIP2+LoRA	Difference
Accuracy	92.78% ± 0.83	71.11% ± 1.26	+21.67% ± 1.51
Average BLEU-4 Score	0.6507 ± 0.0154	0.4501 ± 0.0203	+0.2006 ± 0.0254
Average ROUGE-L Score	0.7184 ± 0.0128	0.5580 ± 0.0185	+0.1604 ± 0.0225
Average Symptom Recall Rate	0.3556 ± 0.0189	0.2833 ± 0.0221	+0.0723 ± 0.0283
Average Inference Latency(ms)	11997.30 ± 186.5	11744.40 ± 152.3	+252.90 ± 240.7

Experimental results demonstrate that the BLAP model achieves substantial improvements in both classification accuracy and text generation quality. Across three independent repeated experiments, the mean crop disease recognition accuracy of BLAP is 92.78% with a standard deviation of 0.83%, representing an average increase of 21.67 percentage points (standard deviation 1.51 percentage points) over the baseline model (71.11% ± 1.26%). In terms of relative improvement over the baseline, the average relative gain is 30.47% ± 2.43%. For text generation quality, BLAP outperforms the baseline by 0.2006 (44.58%) in BLEU-4, by 0.1604 (28.75%) in ROUGE-L, and by 0.0723 (25.52%) in symptom recall.

Two-tailed paired t-tests (α=0.05, n=3) indicate that the differences between BLAP and the baseline are statistically significant across all core evaluation metrics: accuracy (t=24.87, p=0.0016), BLEU-4 (t=13.68, p=0.0053), ROUGE-L (t=12.35, p=0.0065), and symptom recall (t=4.43, p=0.0470), with all p-values below 0.05. These results provide strong evidence that the significant gains of BLAP in classification and text generation are not attributable to random fluctuations, thereby validating the synergistic effectiveness of the multi-scale pyramid feature fusion and adaptive prompt fusion modules. Regarding inference latency, BLAP introduces an average increase of 252.90 ms (approximately 2.15%) over the baseline, yet this difference does not reach statistical significance (t=1.82, p=0.2105), indicating that the incorporation of the fusion modules does not incur a notable additional inference overhead.

While achieving a notable improvement in recognition accuracy, the BLAP model exhibits an average increase in inference time of only 252.90ms (240.7ms standard deviation) compared to the baseline, corresponding to a relative increment of approximately 2.15%. This result indicates that the fusion modules and the LoRA low-rank matrices are successfully merged into the forward pass of the backbone model during inference. It should be noted that the current average inference time is approximately 12 seconds per image. Although this latency remains insufficient for real-time processing on resource-constrained edge devices, it is adequate for practical non-real-time applications, such as offline field diagnosis, asynchronous analysis on expert consultation platforms, and cloud-assisted mobile diagnosis, where users upload images and receive diagnostic results within a short response time. For deployment in real-time monitoring systems, such as unmanned aerial vehicles (UAVs) or agricultural robots, future work will focus on further reducing inference latency through model compression techniques, including post-training 4-bit quantization followed by structured pruning or knowledge distillation, while maintaining diagnostic accuracy.

**Figure 6:**
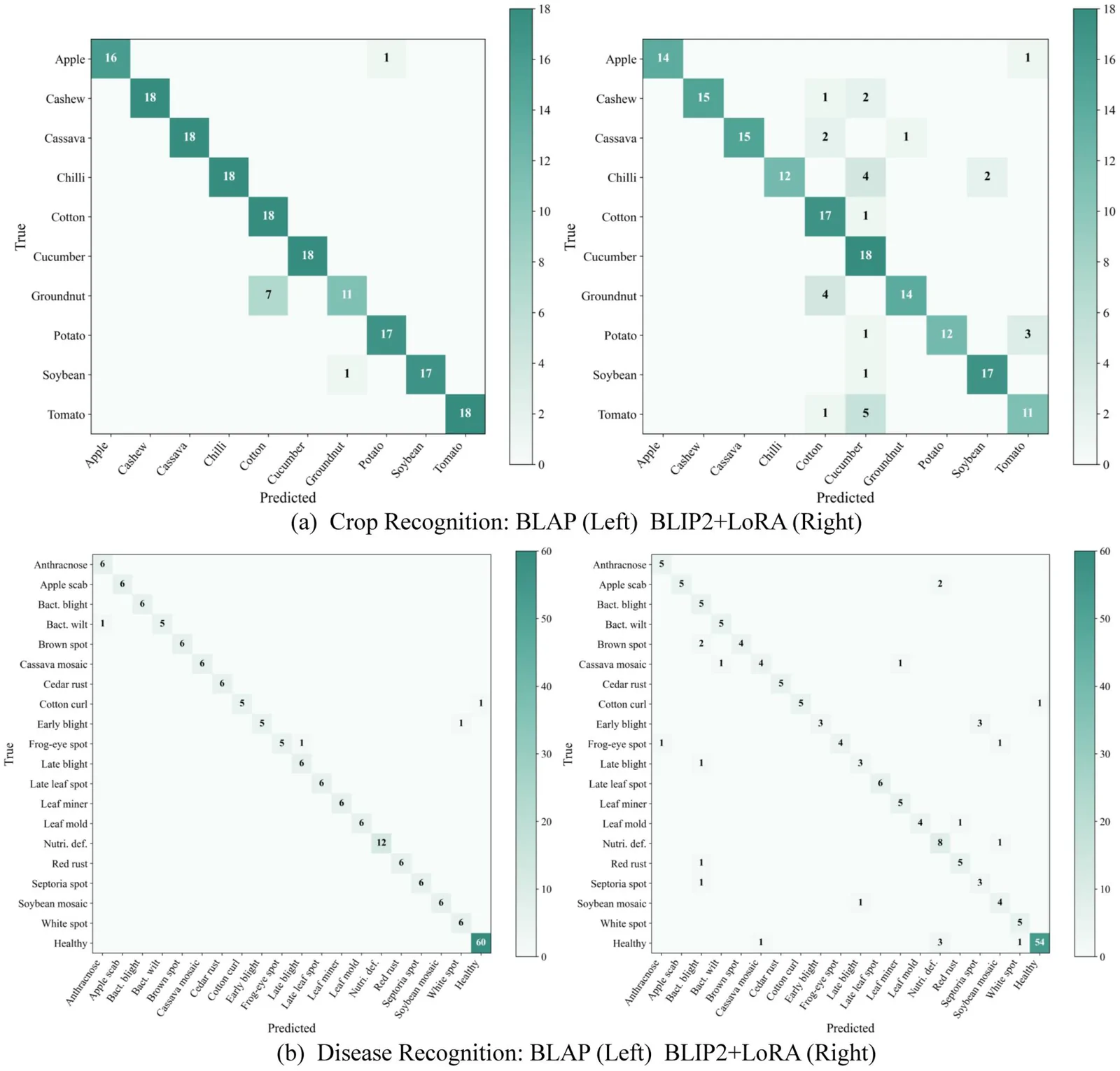
Comparison of confusion matrices between BLAP and BLIP2+LoRA: **(a)** crop recognition, with BLAP on the left and BLIP2+LoRA on the right; **(b)** disease recognition, with BLAP on the left and BLIP2+LoRA on the right.

Comparison of the confusion matrices for crop identification (**Figure 6a**) and disease identification (**Figure 6b**) further reveals that BLAP achieves superior classification performance on most samples, with significantly fewer misclassifications than the BLIP2+LoRA baseline.

### 3.2 Ablation experiment

To comprehensively quantify the individual contributions and synergistic effects of each submodule, we conduct systematic ablation studies based on the BLIP-2+LoRA baseline, denoted as B0. These studies include the following aspects: verifying the independent contributions of the Pyramid Fusion Module (PFM) and the Adaptive Prompt Fusion Module (APFM); validating the standalone role of the gating mechanism; examining the impact of the number of prompts K on performance; confirming the necessity of the LoRA baseline; and analyzing the trade-off between model accuracy and inference latency under different LoRA rank r configurations. The complete experimental results are presented in **Table 4**.

**Table 4:** Ablation study results of the core modules in the proposed BLAP framework.

Experiment ID	LoRA	PFM	APFM	gate	K	LoRA(r)	Accuracy	BLEU-4	ROUGE-L	Recall	Average inference latency (ms)
B11							32.22% ± 2.31	0.1317 ± 0.0196	0.2676 ± 0.0224	0.0167 ± 0.0067	7397.54 ± 102.5
B12		✓	✓	✓	16		0.00% ± 0.00	0.0000 ± 0.0000	0.0078 ± 0.0041	0.0000 ± 0.0000	655.53 ± 8.7
B0	✓					8	71.11% ± 1.26	0.4501 ± 0.0203	0.5580 ± 0.0185	0.2833 ± 0.0221	11744.40 ± 152.3
B1	✓	✓				8	86.67% ± 1.20	0.5770 ± 0.0168	0.6611 ± 0.0153	0.3333 ± 0.0204	20358.88 ± 215.6
B2	✓		✓	✓	16	8	73.33% ± 1.22	0.4629 ± 0.0171	0.5572 ± 0.0148	0.3167 ± 0.0210	12160.89 ± 205.8
B4	✓	✓	✓	1	16	8	88.33% ± 1.08	0.4528 ± 0.0215	0.5628 ± 0.0192	0.3500 ± 0.0198	12535.32 ± 162.7
B5	✓	✓	✓	✓	8	8	92.22% ± 0.94	0.5588 ± 0.0165	0.6524 ± 0.0145	0.3500 ± 0.0205	12295.96 ± 176.3
B3	✓	✓	✓	✓	16	8	92.78% ± 0.83	0.6507 ± 0.0154	0.7184 ± 0.0128	0.3556 ± 0.0189	11997.30 ± 186.5
B6	✓	✓	✓	✓	32	8	87.22% ± 1.15	0.5247 ± 0.0182	0.6252 ± 0.0159	0.3611 ± 0.0193	11902.72 ± 165.8
R4	✓	✓	✓	✓	16	4	49.44% ± 2.89	0.2304 ± 0.0241	0.3513 ± 0.0225	0.2111 ± 0.0263	12661.02 ± 178.9
R16	✓	✓	✓	✓	16	16	94.44% ± 0.88	0.5788 ± 0.0197	0.6676 ± 0.0162	0.3611 ± 0.0208	13406.66 ± 195.7

Experiment B11 shows that the original BLIP-2 model without LoRA or any fusion modules achieves only 32.22% recognition accuracy under few-shot conditions, indicating that generic vision-language models are severely inadequate when applied directly to agricultural disease diagnosis. After introducing LoRA alone (B0), the accuracy improves to 71.11%, demonstrating the effectiveness of parameter-efficient fine-tuning for task transfer. Since the PFM and APFM fusion modules without LoRA adaptation fail to converge stably under the 4-bit quantized BLIP-2 setting (B12), we adopt BLIP-2+LoRA (B0) as the baseline for subsequent ablation comparisons.

**Figure 7:**
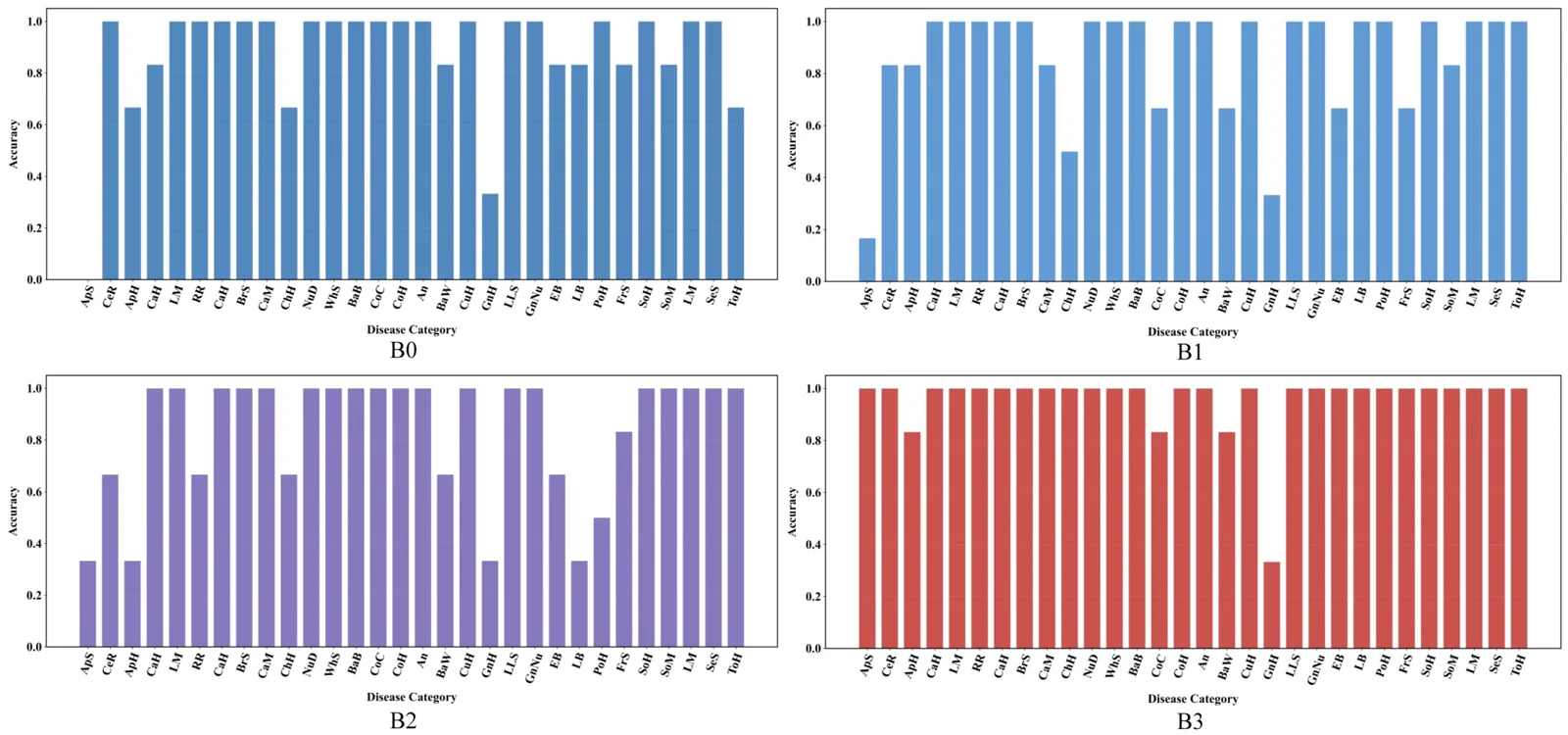
Per-disease accuracy comparison across ablation models.

Comparison between B1 and B0 shows that introducing the Pyramid Fusion Module (PFM) into the standard LoRA fine-tuning pipeline improves model accuracy by 15.56%, whereas adding only the Adaptive Prompt Fusion Module (APFM) as in B2 yields a modest gain of only 2.22%. This indicates that the substantial performance boost of BLAP is mainly attributed to the multi-scale pyramid fusion module, which addresses the core limitation of the original BLIP-2 model, namely its single-scale visual representation. Crop disease features span an extremely wide range of scales, and ordinary LoRA-based optimization strategies are insufficient to capture multi-scale visual characteristics, thereby leading to classification errors. By injecting multi-scale disease visual information into the language model, PFM enables the model to simultaneously capture fine-grained lesion details and coarse-grained foliar pathological changes, thus improving recognition accuracy. Furthermore, **Figure 7** demonstrates that after introducing PFM, the misclassification rate of B1 decreases markedly, which rigorously confirms that the PFM module effectively enhances the capture of multi-scale disease symptoms and improves visual recognition performance. The relatively low recognition accuracy for peanut nutrient deficiency (GnH) can be attributed to the fact that its foliar manifestation consists primarily of chlorosis and yellowing, which are highly overlapping in visual appearance with other conditions and lack distinctive pathological hallmarks, making discrimination considerably more challenging than for other diseases.

**Figure 8:**
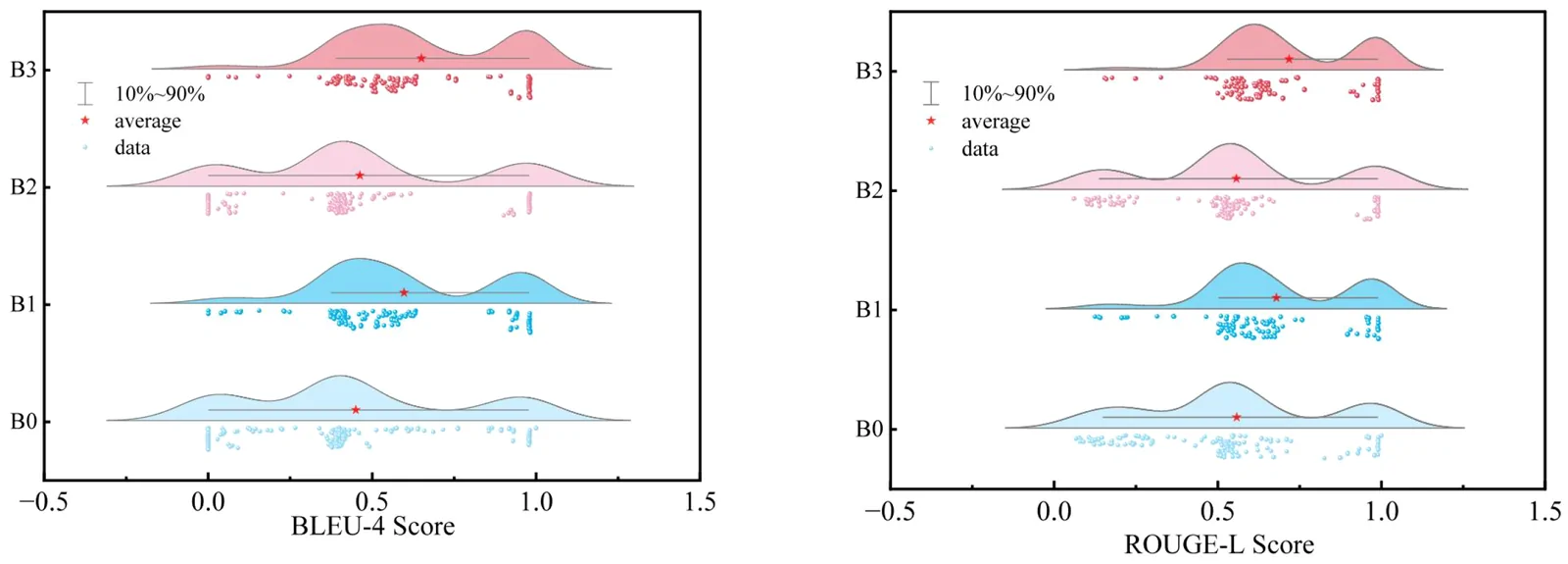
Distribution of BLEU-4 and ROUGE-L across models.

The Adaptive Prompt Fusion Module (APFM) also yields a notable standalone effect. As shown by the comparison between B2 and B0 in **Table 4**, B1, which incorporates only the Pyramid Fusion Module (PFM), exhibits the largest average inference latency at 20358.88ms. This is likely because PFM performs dense multi-scale feature extraction and concatenation in the high-dimensional image space, involving heavy computation without any dynamic sparsity strategy. In contrast, B3, which introduces APFM in addition to PFM, achieves a substantially lower inference overhead. This reduction can be attributed to the fact that APFM shifts feature fusion from the image space to the low-dimensional prompt vector space and employs a gating mechanism for dynamic sparse inference, greatly reducing the computational burden. Moreover, compared with B1 (PFM only), B3 (PFM+APFM) improves the average BLEU-4 and average ROUGE-L by 7.37% and 5.73%, respectively (**Figure 8**), confirming that APFM effectively complements PFM by guiding the model to generate higher-quality diagnostic text conditioned on visual information.

In **Table 4**, B4, where the gating values are frozen to a constant of 1 (i.e., unconditional and full injection of visual information), exhibits degraded performance relative to the complete BLAP model (B3). This further demonstrates that the adaptive gating mechanism, by dynamically modulating the injection intensity, effectively prevents irrelevant visual information from interfering with text generation. In particular, when generating content that relies heavily on linguistic priors, such as treatment recommendations, the model can adaptively reduce the visual injection strength, thereby preserving the semantic plausibility of the generated text.

Integrating PFM and APFM sequentially into BLIP-2+LoRA yields the complete BLAP model (B3), which achieves the best recognition accuracy, confirming the overall rationality and effectiveness of the proposed design. Moreover, as shown in **Figure 8**, BLAP attains the highest ROUGE-L and BLEU-4 scores among all methods, substantially outperforming the other models. This indicates that BLAP generates outputs that align more closely with the reference texts in terms of lexical choice and phrase order, demonstrating superior language generation quality. In addition, BLAP exhibits a clear advantage in both lexical overlap and sequential consistency with the reference content, enabling better capture of key information from the reference summaries. Notably, the average inference latency of BLAP increases by only 252.9ms, which is approximately 2.15% of the baseline latency, a margin that is fully acceptable for practical agricultural applications. Experiments B5, B3, and B6 correspond to K = 8, 16, and 32, respectively. The results show that K = 16 yields the best performance, which is the BLAP configuration. With K = 8, the visual prompt capacity is insufficient to cover the diverse disease visual patterns, whereas with K = 32, redundant information is introduced along with increased computational cost. This validates the choice of 16 prompt vectors in this work.

Three configurations with LoRA ranks r = 4, 8, and 16 are compared, corresponding to R4, B3, and R16, respectively. As shown in **Table 4**, the rank has a significant impact on model performance. At r=4, the recognition accuracy is 49.44%, and BLEU-4 and ROUGE-L are 0.2304 and 0.3513, respectively, all substantially lower than those of the full BLAP model (r=8) at 92.78%, 0.6507, and 0.7184. This indicates that an excessively low rank limits the representational capacity of the low-rank decomposition matrices, making it difficult to capture the complex feature patterns in crop disease recognition. At r=16, the accuracy further improves to 94.44%, a gain of 1.66% over r=8, but BLEU-4 and ROUGE-L drop to 0.5788 and 0.6676, respectively, which are lower than those at r=8. This suggests that while a higher rank (r=16) brings a modest gain in classification, it leads to a notable degradation in text generation quality. In terms of parameter counts, r=4, r=8, and r=16 correspond to approximately 0.49 million, 0.98 million, and 1.97 million trainable LoRA parameters, respectively. At r=4, the model capacity is insufficient, posing a higher risk of underfitting; at r=16, while capacity is ample, overfitting is more likely under few-shot conditions, impairing the generalization of generated text. Overall, r=8 achieves the best trade-off, justifying the choice of LoRA rank r=8 in this work.

To verify the effectiveness of the proposed lesion-aware mechanism, we conduct a visual analysis of the model’s attended regions using Grad-CAM. Four representative correctly classified samples are selected for illustration, namely Apple scab, Cedar apple rust, Brown leaf spot, and Cassava mosaic disease.

**Figure 9:**
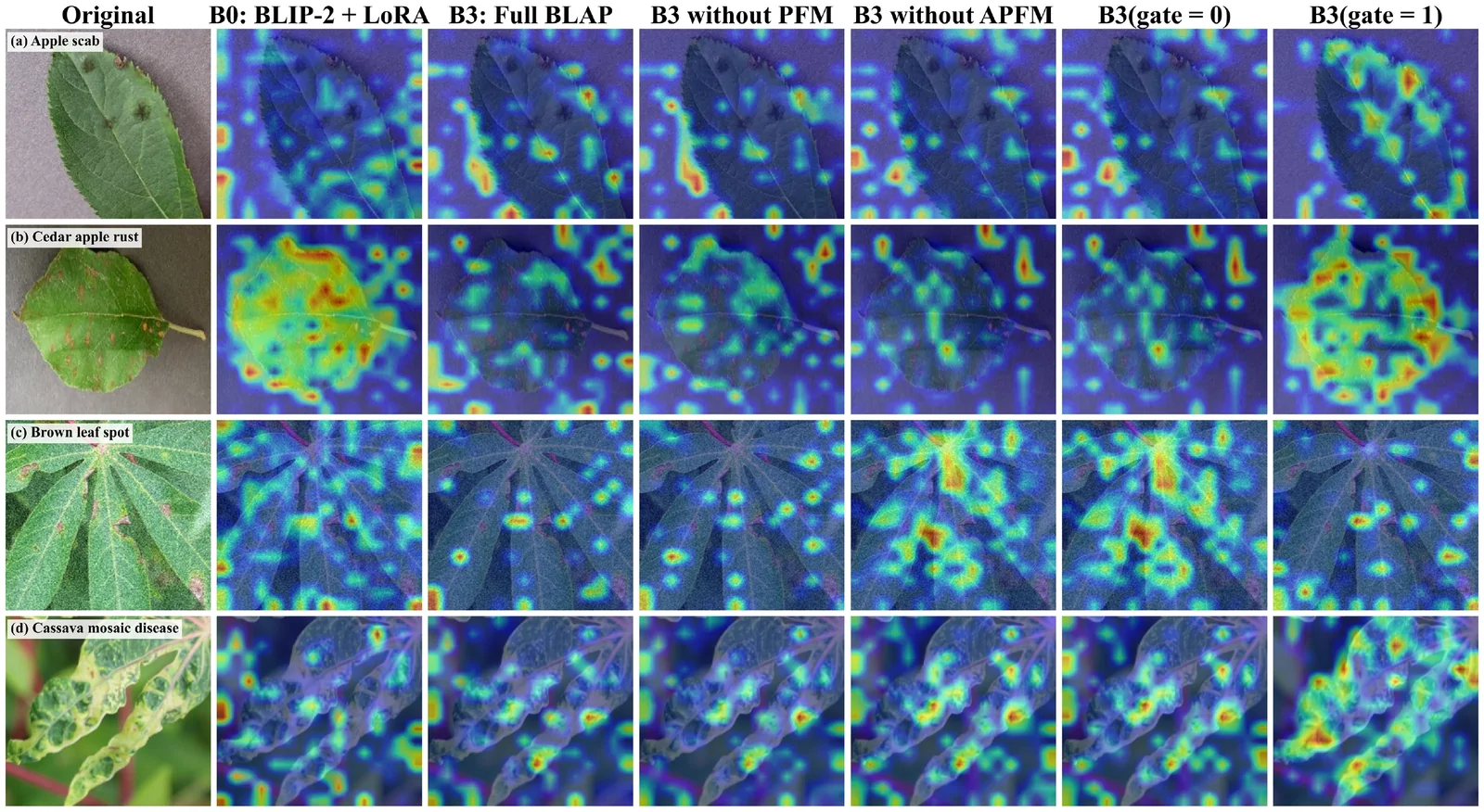
Grad-CAM visualizations of representative crop disease samples: **(a)** apple scab, **(b)** cedar apple rust, **(c)** brown leaf spot, and **(d)** cassava mosaic disease. Columns compare the original image, BLIP-2+LoRA, full BLAP, BLAP without PFM, BLAP without APFM, and BLAP with gate fixed at 0 or 1.

As shown in **Figure 9**, compared with the baseline BLIP-2+LoRA, the full BLAP model exhibits stronger focus on disease-relevant regions across most samples. Its high-response areas are more concentrated on lesion spots, rust spots, brown spots, and mosaic symptoms, while responses to irrelevant backgrounds and healthy leaf tissues are relatively suppressed. Furthermore, when either the pyramid fusion module (without PFM) or the adaptive prompt fusion module (without APFM) is removed, the attention distributions become dispersed or shifted to varying degrees, and the consistency of responses over disease-relevant regions decreases. When the gating is fixed to extreme states, particularly with gate=1, the heatmaps tend to exhibit excessive expansion, indicating that relying solely on strong visual injection introduces more non-discriminative responses. In contrast, the full BLAP model, through the synergistic effect of multi-scale pyramid representations and dynamic gating, preserves critical disease information while suppressing irrelevant interference, thereby enhancing the model’s representation capability for disease-related regions, which is consistent with its improved classification performance.

**Figure 10:**
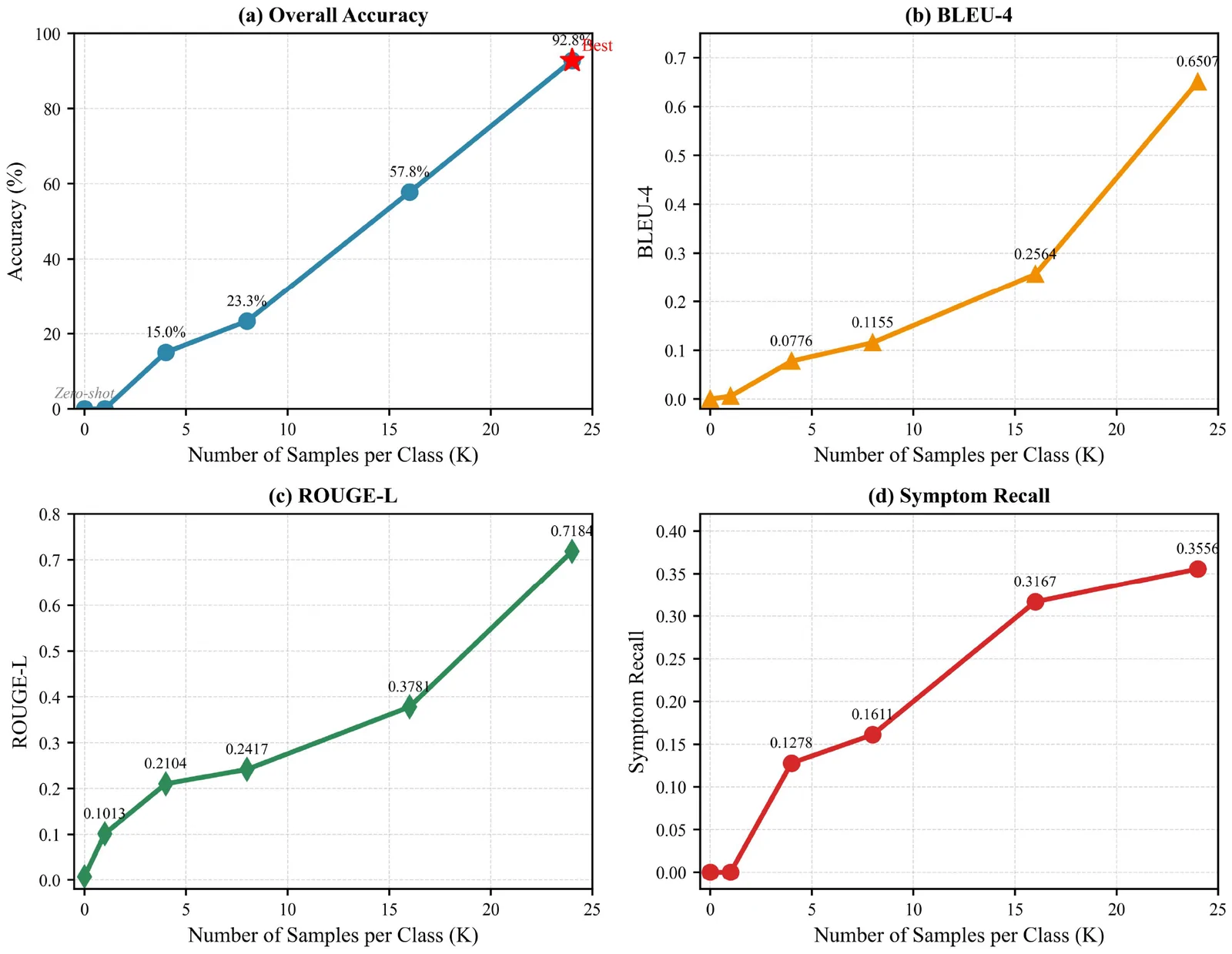
Performance of BLAP under different numbers of training samples per class: **(a)** overall accuracy, **(b)** BLEU-4, **(c)** ROUGE-L, and **(d)** symptom recall.

In addition, to evaluate the adaptability and data efficiency of the BLAP model under extremely low annotation cost, we conduct a sample size ablation experiment. Specifically, from each category of the training set, we randomly draw K={0,1,4,8,16,24} samples for independent training and evaluate the model performance on the same test set, where K = 0 denotes zero-shot inference without any fine-tuning. **Table 5** summarizes the key evaluation metrics under different values of K, and **Figure 10** presents the trend of each metric as the sample size varies.

**Table 5:** Performance comparison of the BLAP model under different sample sizes.

Samples per class (K)	Zero-shot	1	4	8	16	24(BLAP)
Accuracy (%)	0.00%	0.00%	15.00%	23.33%	57.78%	92.78%
BLEU-4	0.0000	0.0057	0.0776	0.1155	0.2564	0.6507
ROUGE-L	0.0078	0.1013	0.2104	0.2417	0.3781	0.7184
Recall	0.0000	0.0000	0.1278	0.1611	0.3167	0.3556

The experimental results demonstrate a strong positive correlation between BLAP performance and the sample size. Under extreme few-shot conditions (K ≤ 8), the model performance increases only slowly, with disease recognition accuracy rising from 0% to 23.33%. This is likely because an extremely limited number of samples is insufficient for the model to effectively capture the discriminative visual features of each disease category. However, when the sample size exceeds a threshold at K = 16, the model performance exhibits a marked jump: recognition accuracy substantially improves from 23.33% to 57.78% (a gain of 34.45 percentage points), while BLEU-4 and ROUGE-L increase by 0.141 and 0.136, respectively. This suggests that once the accumulated samples reach a certain level, the multi-scale feature fusion module and the adaptive prompt module within the BLAP framework can be effectively activated, collaboratively establishing more discriminative class prototypes and semantic alignments. As the sample size further increases to K = 24 (the standard BLAP setting), all metrics continue to improve, achieving an accuracy of 92.78%, a BLEU-4 of 0.6507, a ROUGE-L of 0.7184, and a symptom recall of 0.3556.

The above analysis indicates that the BLAP framework can achieve considerable disease diagnosis performance under very low annotation cost (16 samples per class), significantly alleviating the bottleneck of scarce labeled samples in the agricultural domain. Nevertheless, the current results also reveal a limitation that warrants attention: when K ≤ 8, the model performance remains nearly at the random-guess level (accuracy ≤23.33%). This is not a defect inherent to the BLAP framework, but rather a common challenge faced by VLMs in ultra-low-resource scenarios, where the model fails to establish effective alignment between visual features and disease category names. Even so, the performance jump at K = 16 demonstrates the strong adaptability of BLAP in moderately label-scarce settings and provides a valuable baseline reference for future research on more efficient ultra-low-resource vision-language alignment strategies. Based on the comprehensive experimental results, all subsequent experiments in this work adopt the standard setting of 24 samples per class to maintain a favorable balance between diagnostic quality and training cost.

**Figure 11:**
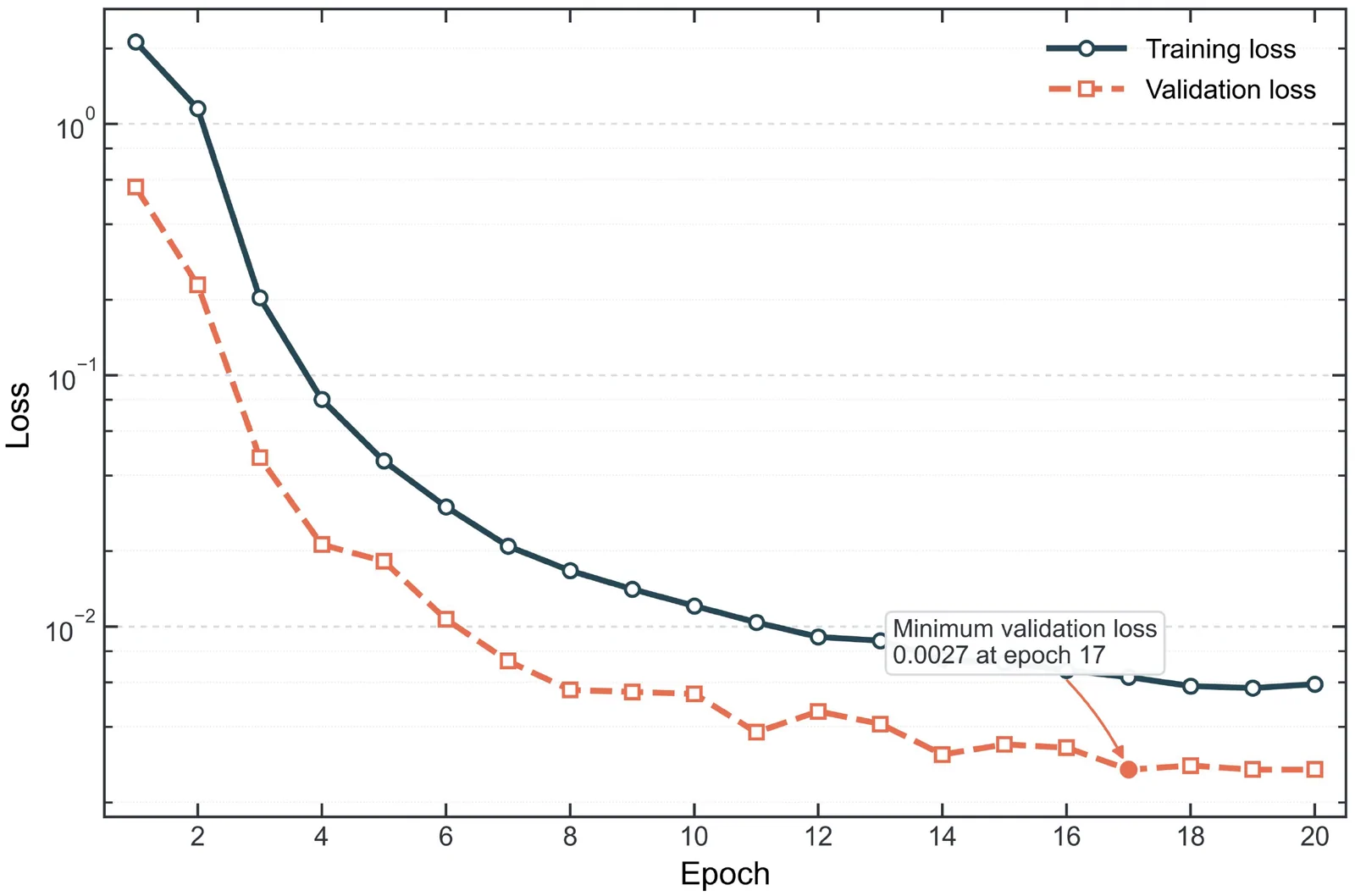
Variation curves of training loss and validation loss of BLAP.

In addition to the above experiments, to verify the training stability and generalization capability of BLAP under limited data, **Figure 11** presents the training and validation loss curves over 20 training epochs.

Overall, the training loss declines rapidly from 2.1219 at epoch 1 to 1.1511 at epoch 2, then continues to decrease steadily and eventually converges to 0.0059 at epoch 20. The validation loss decreases from 0.5613 at epoch 1 and reaches its minimum value of 0.0027 at epoch 17, and remains consistently lower than the training loss throughout the entire process, indicating that the model does not suffer from notable overfitting. Notably, after epoch 11, both training and validation losses stabilize below 0.006, and the best model, selected based on the lowest validation loss, is saved at epoch 11. This convergence behavior benefits from the multiple regularization strategies adopted in our framework: the LoRA low-rank constraint with r=8 inherently restricts the degrees of freedom in the parameter space, serving as structural regularization; the 4-bit NF4 quantization introduces parameter noise with an implicit regularizing effect; dropout at a rate of 0.1 is applied in both feature projection layers and attention modules; the AdamW optimizer is used with weight decay of 0.01; gradient clipping with a maximum norm of 1.0 prevents gradient explosion; the learning rate schedule combines a warmup over 10% of the total steps with cosine annealing; and online data augmentation, including random resized cropping, horizontal flipping, rotation, and color jitter, is employed during training to effectively increase the diversity of the limited samples. The synergy of these strategies ensures that BLAP converges stably and maintains strong generalization despite having only 24 training samples per class.

### 3.3 Comparative experiments with other models

To comprehensively evaluate the performance advantages of BLAP in crop disease diagnosis, we compare against a diverse set of representative baseline models spanning both discriminative and generative paradigms. These include classical CNN image classifiers as well as general-purpose and agriculture-domain vision-language models. Specifically, we evaluate ResNet50, a convolutional neural network widely adopted for plant pathology image classification, which we fine-tune in full on the same training set. We also assess AgriCLIP, a vision-language model pre-trained for the agricultural domain, under two protocols: official zero-shot inference and linear probing where the backbone is frozen and only the classification head is trained. In addition, we test the zero-shot classification performance of SCOLD, a recently proposed domain-specific vision-language model for leaf disease recognition. To enable generative diagnosis with SCOLD, we attach a lightweight visual prefix projector after its frozen image encoder, adopt the same OPT-2.7B language model and LoRA adaptation strategy as in BLAP, and train and evaluate the resulting model, denoted as SCOLD-OPT-Adapter, under identical data splits. Furthermore, we include BLIP-2 in its original frozen form without any parameter update as a zero-shot baseline, and BLIP-2 with LoRA fine-tuning only, which serves as the primary baseline for comparison in this work.

All models are either trained or evaluated on the same training set of 792 images (24 per class) and test set of 198 images (33 classes) to ensure fair comparison. For models that only support classification output, we report recognition accuracy and macro-average F1 score. For generative models, we additionally report BLEU-4, ROUGE-L, and symptom recall to comprehensively assess the quality of diagnostic text. **Table 6** summarizes the comparative experimental results.

**Table 6:** Performance comparison of different models on plant disease recognition task.

Model	Accuracy (%)	Macro-F1	BLEU-4	ROUGE-L	Symptom recall rate
ResNet50	98.99 ± 0.31	0.9896 ± 0.0035			
AgriCLIP (zero-shot)	2.53 ± 0.89	0.0108 ± 0.0051			
AgriCLIP	49.49 ± 2.10	0.4489 ± 0.0283			
SCOLD (zero-shot)	61.11 ± 1.73	0.5076 ± 0.0256			
SCOLD-OPT-Adapter	94.33 ± 0.76	0.9826 ± 0.0042	0.6349 ± 0.0162	0.6880 ± 0.0135	0.3611 ± 0.0205
BLIP2 (zero-shot)	32.22 ± 2.31	0.3056 ± 0.0287	0.1317 ± 0.0196	0.2676 ± 0.0224	0.0167 ± 0.0067
BLIP2+ LoRA	71.11 ± 1.26	0.7612 ± 0.0184	0.4501 ± 0.0203	0.5580 ± 0.0185	0.2833 ± 0.0221
BLAP(ours)	92.78 ± 0.83	0.9778 ± 0.0053	0.6507 ± 0.0154	0.7184 ± 0.0128	0.3556 ± 0.0189

**Table 6** shows that ResNet50 achieves the highest classification accuracy at 98.99% and macro-average F1 at 0.9896, which is expected given that it is fully fine-tuned on the entire training set with a moderate model size. However, ResNet50 is restricted to closed-set category labels and cannot provide any symptom descriptions or treatment recommendations, thus serving only as a simple discriminative tool in practice, with far less informativeness and interactivity than BLAP. Notably, BLAP, while updating only 0.11% of the total parameters, lags behind ResNet50 by merely 6.21 percentage points in accuracy, yet it generates semantically coherent and actionable diagnostic reports, offering substantially higher practical value. Although AgriCLIP is pre-trained on agricultural data, its zero-shot inference accuracy is only 2.53% and macro-average F1 is 0.0108, indicating a severe semantic gap between its visual encoder and our 33-class disease label space. Even with linear probing for light adaptation, the accuracy improves to only 49.49%, still far below BLAP. This strongly suggests that neither domain-specific pre-training nor shallow adaptation alone is sufficient for fine-grained, multi-scale disease recognition, and the multi-scale pyramid feature fusion (PFM) and adaptive prompt fusion (APFM) introduced in BLAP exactly address this deficiency.

Among all generative models, BLAP leads across all metrics. Compared with the BLIP-2+LoRA baseline, BLAP improves accuracy by 21.67 percentage points, BLEU-4 by 20.06%, ROUGE-L by 16.04%, and symptom recall by 7.23%. These gains directly reflect the synergistic effect of PFM and APFM: PFM injects multi-scale lesion visual cues, while APFM dynamically modulates the visual guidance strength according to the generated content, jointly enhancing both classification precision and generation quality. Notably, BLAP even surpasses the domain-specific model SCOLD-OPT-Adapter. The latter, pre-trained on a large scale with 186,000 agricultural image-text pairs, achieves a slightly higher classification accuracy of 94.33% than BLAP, but its BLEU-4 of 0.6349 and ROUGE-L of 0.6880 are both lower than those of BLAP, and its symptom recall of 0.3611 is only marginally better than BLAP’s 0.3556. This indicates that BLAP exhibits stronger advantages in phrase collocation, sequential consistency, and coverage of key information in generated text. Considering that SCOLD-OPT-Adapter requires massive pre-training corpora and expensive computational resources, whereas BLAP relies only on a general-purpose BLIP-2 model and a minimal number of labeled samples to achieve comparable or even superior generation quality, our parameter-efficient fine-tuning strategy demonstrates significant competitiveness in both cost and performance. In addition, the original BLIP-2 zero-shot model performs poorly in generation quality, with BLEU-4 of only 0.1317 and symptom recall of 0.0167, confirming that a generic VLM without adaptation is inadequate for such specialized diagnostic tasks.

**Figure 12:**
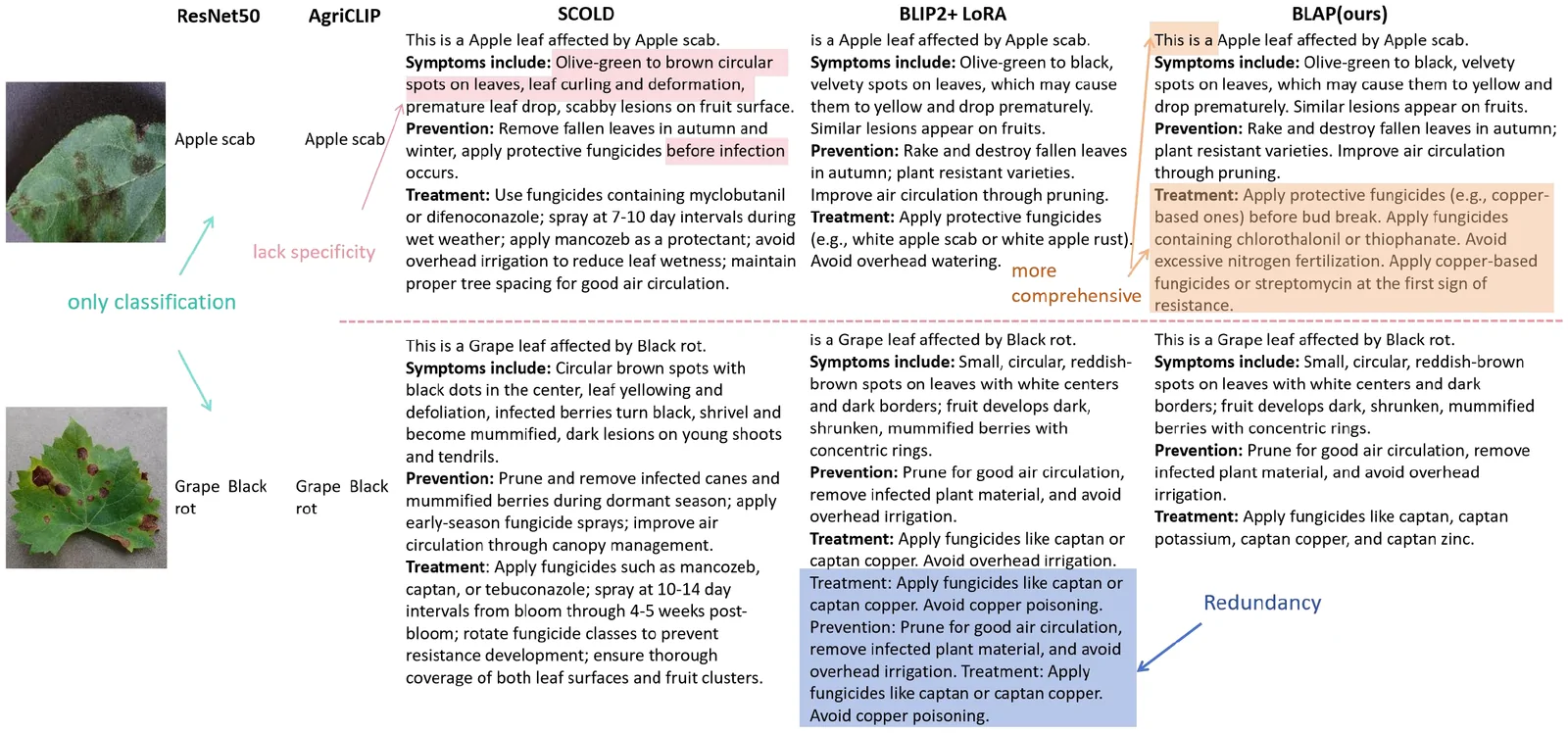
Comparison of generated diagnostic text quality between different models.

To provide an intuitive comparison of generation quality, we randomly select two samples from the test set and juxtapose the outputs of all generative models in **Figure 12**. The text generated by BLIP-2+LoRA frequently suffers from repeated phrases or incomplete sentences. SCOLD-OPT-Adapter, while producing fluent and non-redundant text, tends to generate overly general descriptions, for example referring to multiple distinct diseases merely as lesions on leaves, without providing specific characterizations of particular symptoms such as the color of rust spore pustules or the shape of necrotic lesions. In contrast, the reports produced by BLAP are well-structured and informative. They not only accurately identify the disease name, but also provide detailed descriptions of lesion color, distribution, and typical morphology, along with targeted preventive measures and treatment recommendations, thus effectively addressing the practical need of agricultural producers for actionable information.

In summary, BLAP achieves the best balance among classification accuracy, text generation quality, parameter efficiency, and inference speed among all compared methods. It not only outperforms the general-purpose baseline BLIP-2+LoRA across all generative metrics, but also matches the domain-specific model SCOLD-OPT-Adapter, which is pre-trained at substantial cost, on multiple generation indicators. These results collectively validate the effectiveness and superiority of the adaptive multi-scale visual prompt fine-tuning paradigm.

### 3.4 Construction of a crop disease diagnosis platform

**Figure 13:**
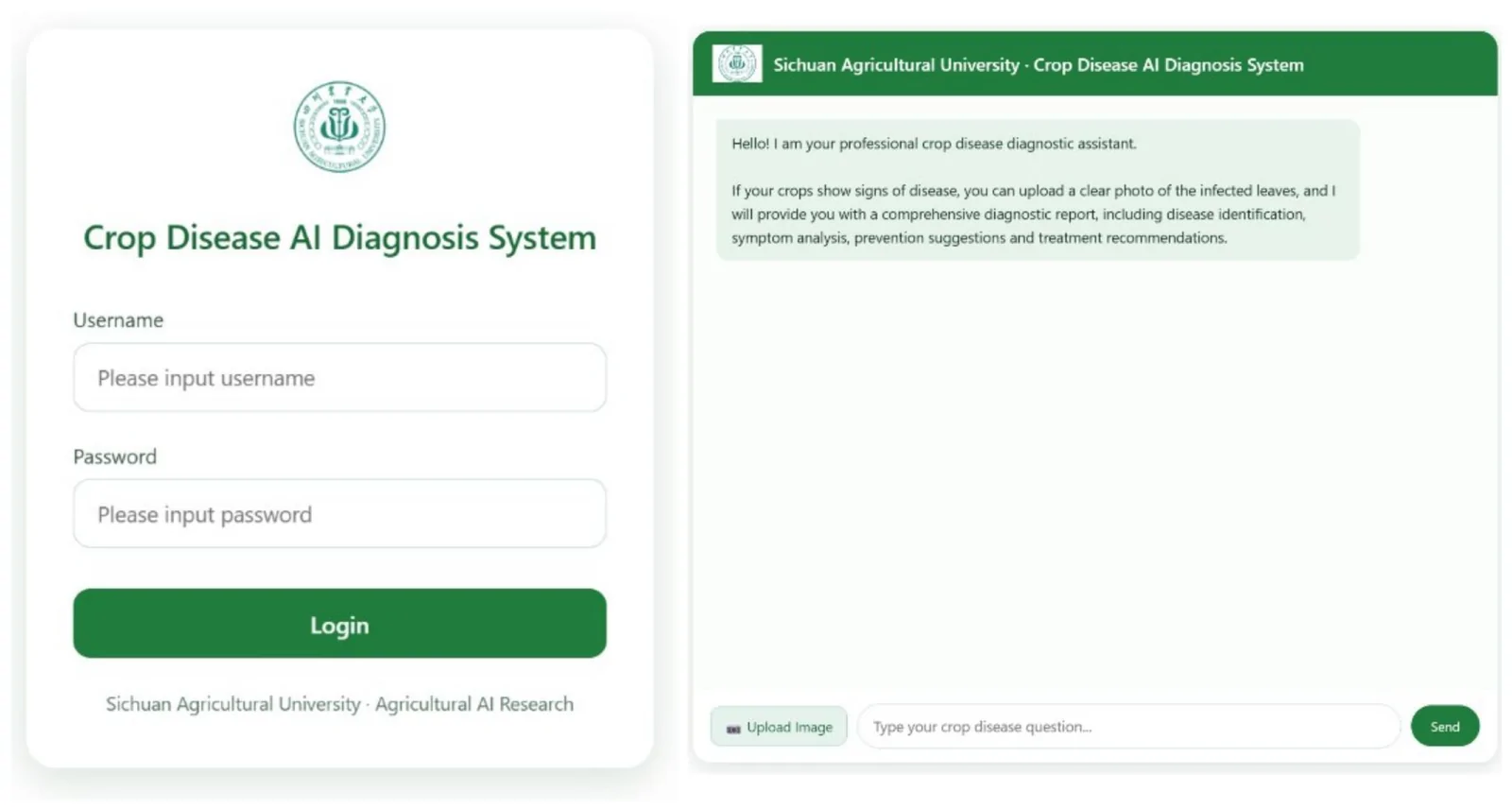
Platform interface for intelligent crop disease diagnosis.

To enhance the practical deployability of the model, this study develops a visualization software platform called the Intelligent Crop Disease Diagnosis Platform based on BLAP, with its interface shown in **Figure 13**. Users only need to take a picture of a crop leaf showing suspected disease symptoms in an actual field, upload the image to the platform, and the model performs crop disease diagnosis, providing outputs including disease description, treatment recommendations, and current management measures.

**Figure 14:**
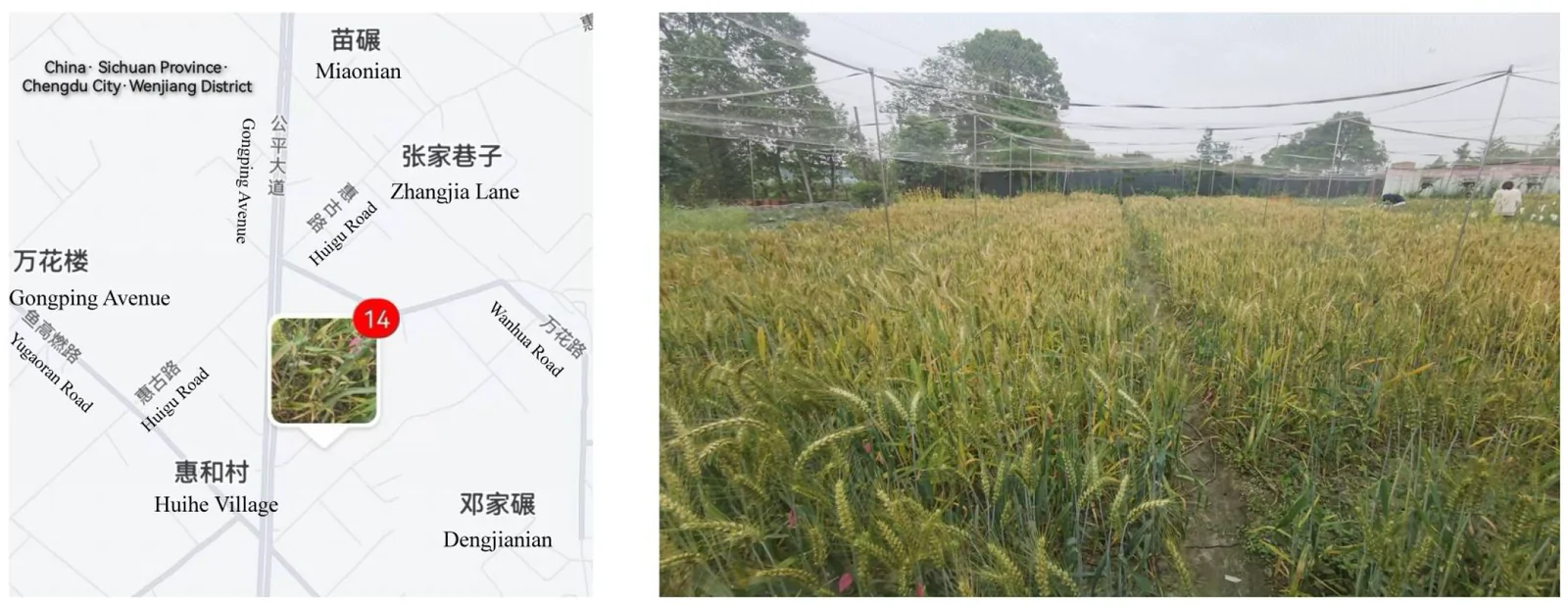
Data collection location (Sichuan Agricultural University Experimental Base).

**Figure 15:**
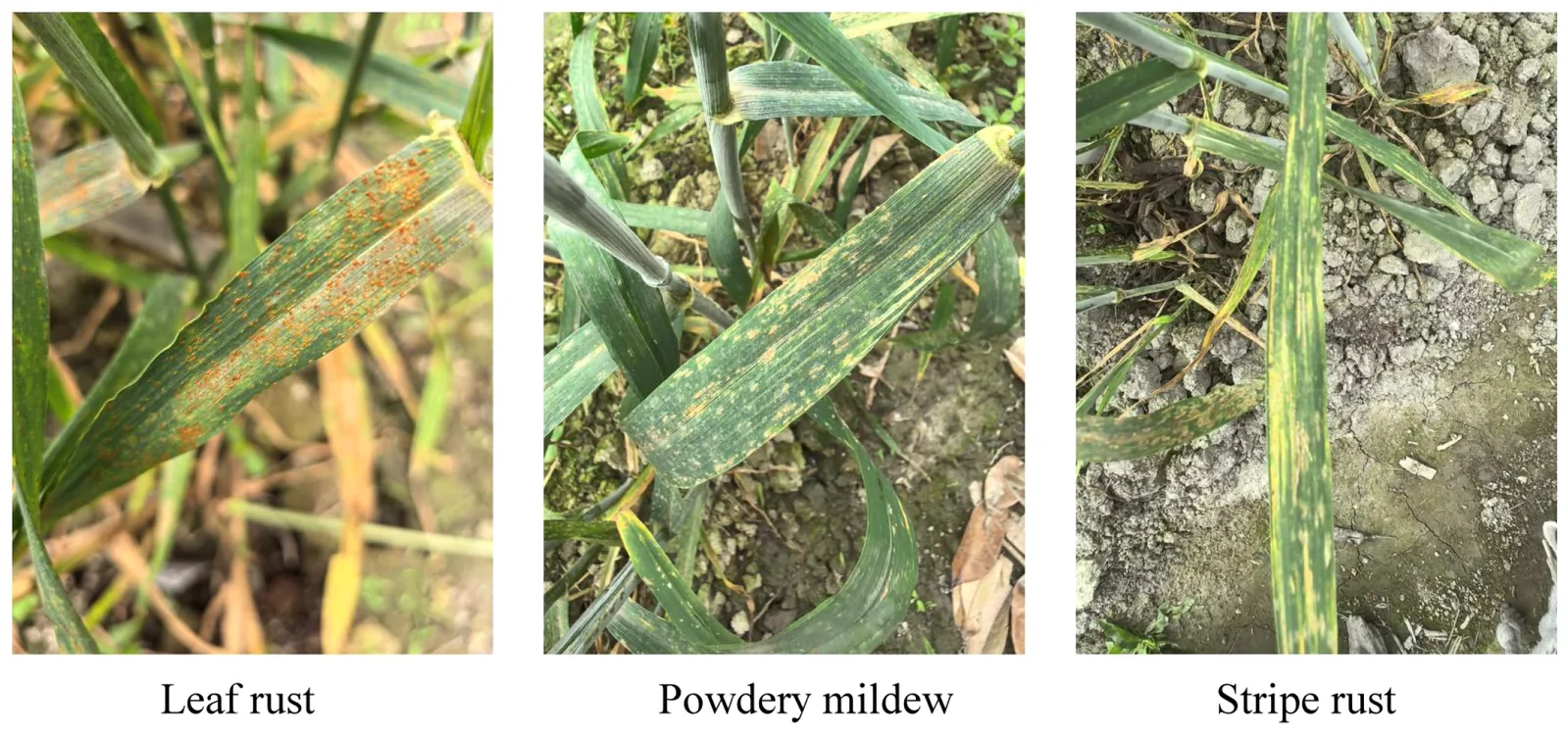
Examples of privately collected wheat disease dataset images.

To evaluate the adaptability of the proposed BLAP framework to previously unseen crop species, we deliberately selected wheat, which was not included in either the training or test datasets used in the main experiments. Images of wheat leaf rust, stripe rust, and powdery mildew were collected under real field conditions to construct an additional dataset. The model was then independently fine-tuned in a few-shot setting using this dataset and subsequently deployed in the developed intelligent crop disease diagnosis platform for evaluation. It should be emphasized that this experiment was designed to demonstrate the few-shot cross-crop adaptation capability of BLAP rather than its zero-shot cross-crop generalization ability. In practical applications, when a new crop species needs to be diagnosed, users only need to provide a small number of annotated images of the target crop to rapidly adapt the model, eliminating the need for training from scratch or full-parameter fine-tuning. This privately collected wheat disease dataset consists of real field-collected wheat samples, with 108 training images and 12 test images for each of the three disease categories, totaling 360 images. The images are captured at the experimental base of Sichuan Agricultural University (103.86°E, 30.71°N; **Figure 14**) with a resolution of 1280×1707 pixels. Sample images from this dataset are shown in **Figure 15**.

**Figure 16:**
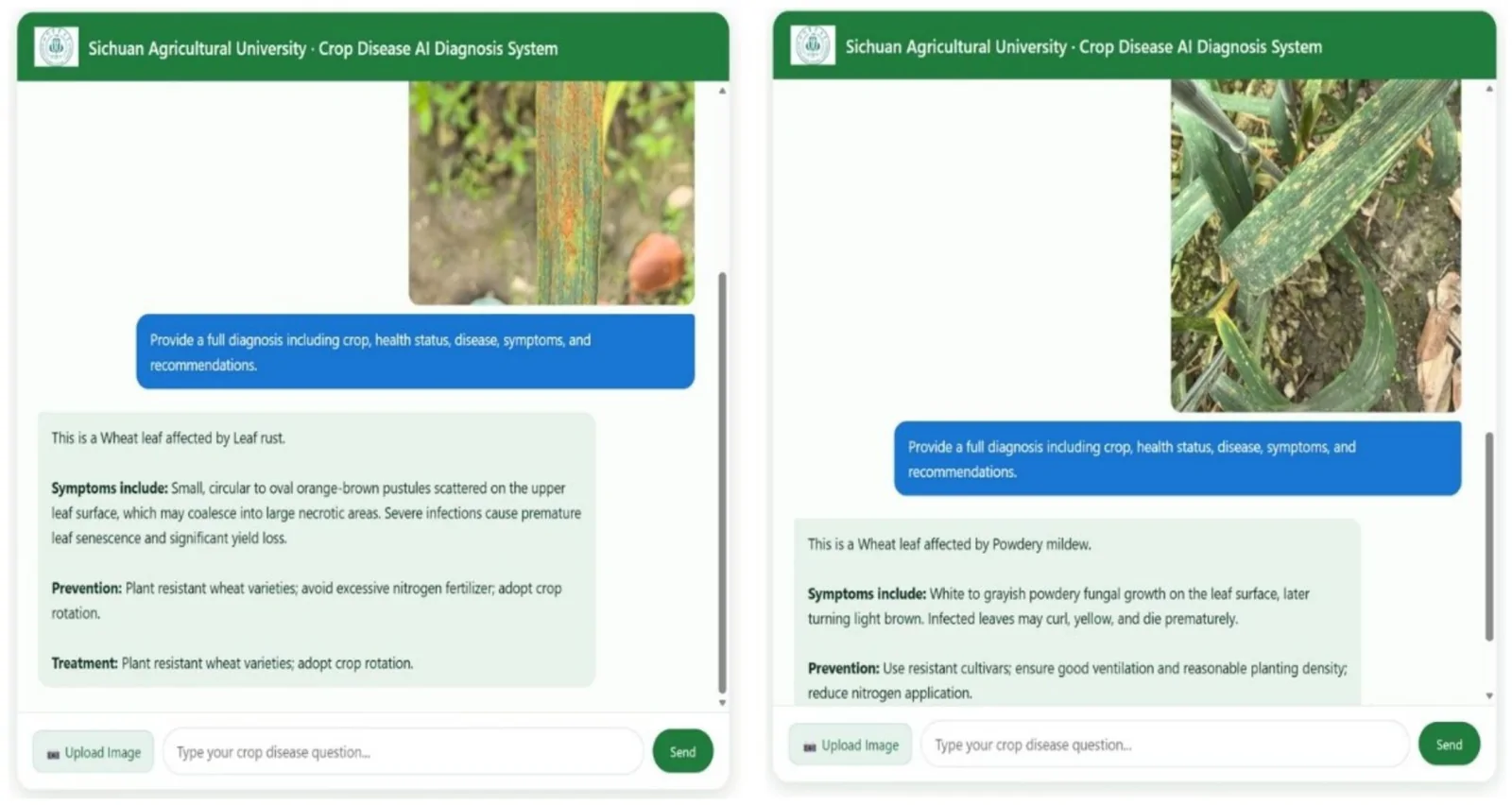
Application effect of the intelligent crop disease diagnosis platform.

**Figure 16** shows the application effect of the intelligent crop disease diagnosis platform. When presented with images captured from real field environments and user questions, the model accurately identifies the disease type and produces complete textual descriptions covering disease symptoms and treatment recommendations. The platform is fast and convenient, can be deployed on mobile devices, and provides strong practical guidance for agricultural production.

**Figure 17:**
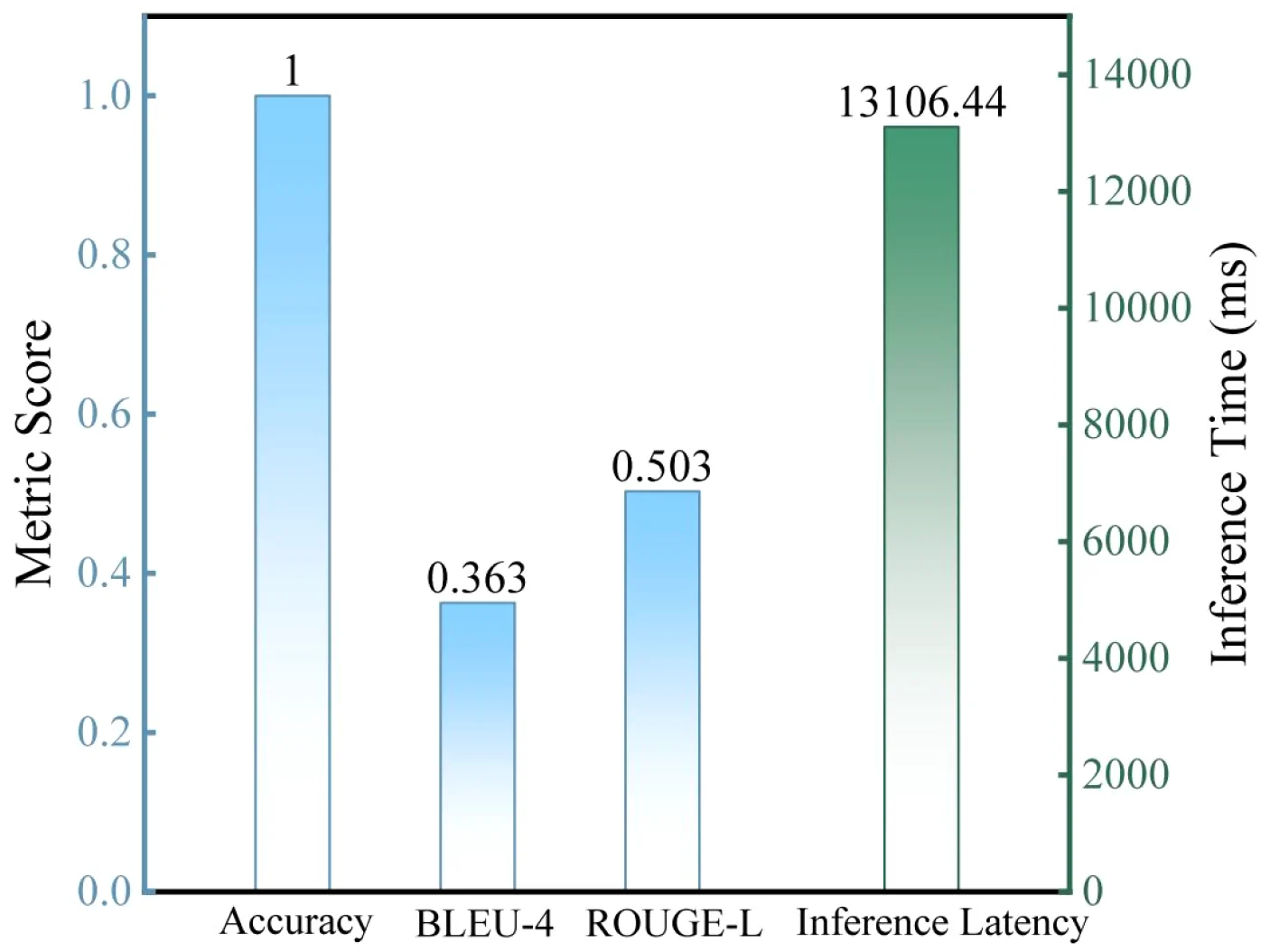
Performance of BLAP on the supplemented dataset.

**Figure 17** presents the test results of BLAP on the entire test set of the privately collected wheat disease dataset (36 images).Although real-world field-collected datasets contain more complex backgrounds and significantly increased inference difficulty, BLAP after few-shot fine-tuning exhibits favorable transferability. All 36 test samples are correctly identified in terms of crop and disease. The model achieves an average BLEU-4 score of 0.3630 and an average ROUGE-L score of 0.5030, indicating that the generated text fully describes key symptom information and provides effective recommendations. In terms of inference efficiency, the average latency per image is 13106.44 ms, which is entirely acceptable for practical applications. Future work can further optimize inference speed through techniques such as model quantization to better meet real-time requirements.

## 4 Discussion

### 4.1 Comparison with related studies

Most existing agricultural disease diagnosis systems adopt a two-model pipeline that separates visual classification from text generation. For instance, the TLDVLM framework proposed by Kaur et al. (2025) employs a multi-stage architecture of front-end segmentation followed by back-end classification, sequentially calling three models: GroundingDINO for object detection, SAM-2 for pixel-level segmentation, and a fine-tuned BLIP-2 for classification. When applied to agricultural tasks, it achieves a recognition accuracy of 97.27% for ten tomato diseases. Similarly, the dual-model architecture of CDIP-ChatGLM3 (Yan et al., 2025) combines computer vision for image-based disease detection with a large language model and demonstrates strong performance in disease recognition and generating actionable prescriptions. Such multi-model designs introduce high complexity and inference latency, as each additional model incurs extra computational overhead and memory footprint. In contrast, the proposed BLAP adopts a fully single-model architecture in which all functionalities are integrated within a single forward pass, requiring no external models, thereby markedly reducing deployment complexity and inference latency.

Recent few-shot disease studies using vision-language models (VLMs) have largely focused on closed-set classification rather than generative full diagnosis. VLCD (Zhou et al., 2024)leverages Qwen-VL to generate image-aware prompts and combines cross-attention with SE attention to improve few-shot classification accuracy; however, it outputs only disease category labels and cannot provide symptom descriptions or control recommendations. Similarly, the adaptively fine-tuned ensemble vision-language model (Yan et al., 2026) introduces an adaptive weighted contrastive loss and an instance-level ensemble strategy to enhance the discrimination of similar diseases, yet it remains a conventional discriminative model that can only perform category prediction. DTM-Unet (Shi et al., 2025) adopts typical pixel-level semantic segmentation, using a hybrid encoder of DenseNet and Vision Transformer with multiple residual atrous convolution modules to accurately delineate lesion areas on cucumber leaves; its core objective is to quantify lesion area for severity assessment. This paradigm assumes that the disease category is known or can be easily classified, and its strength lies in high localization accuracy (pixel accuracy reaching 98.71%), but the output is a binary mask without semantic description capability. The improved architecture EBGC-RTDTR based on RT-DETR-r18 (Huo et al., 2025) achieves both performance enhancement and lightweight design, yet it is also confined to disease category detection and only addresses a single crop, eggplant. Venkatraman et al. (2025) proposed SPROUT, a few-shot precision agriculture framework that employs a symptom-centric prototype optimization mechanism; it uses an attention mechanism to weight support set samples and constructs discriminative class prototypes for metric-based classification (Snell et al., 2017). Under a ten-training-sample setting on rice, sugarcane, cassava, and other crops, it reaches recognition accuracies of 90%--96%, outperforming traditional CNN methods by 11–29 percentage points. Although SPROUT’s prototype optimization is carefully designed, its output remains a closed-set class label and cannot offer more informative details such as symptom manifestations or control strategies. This study redefines disease diagnosis as a generative visual question answering (VQA) task, where the model must not only identify the disease but also describe its visual symptoms and propose reasonable control measures. This paradigm shift elevates BLAP from a mere disease recognition classifier to an interactive diagnostic assistant through the intelligent crop disease diagnosis platform developed in this study.

Building a specialized vision-language model typically incurs high costs. The innovative fine-tuning strategy proposed in this paper incurs substantially lower cost. SCOLD, introduced by Quoc et al. (2025), is a foundational vision-language model for leaf disease recognition trained from scratch. Leveraging a dedicated large-scale dataset of 186,000 agriculture-specific image-text pairs, SCOLD outperforms general-purpose VLMs such as CLIP, LLaVA, and Qwen-VL on both zero-shot and few-shot classification tasks. As an exemplar of domain-specific pretraining, its high performance depends on massive, high-quality, meticulously annotated data and enormous pretraining computation; subsequent iterative updates are also complex and expensive. To reduce model training costs, we propose an innovative fine-tuning strategy for vision-language models. Built upon the off-the-shelf open-source BLIP-2 framework, this strategy introduces a small amount of domain-specific data and local parameter fine-tuning to efficiently accomplish the objective at minimal expense. Moreover, BLAP can benefit from future iterations of the underlying model for further upgrades, leading to sustained savings. This pronounced low-cost advantage facilitates the practical deployment and widespread adoption of the BLAP model in production environments.

Generalization and transferability determine the practical applicability of a model in production settings. TLDVLM (Kaur et al., 2025) performs well on tomato leaf disease discrimination by leveraging the detection and segmentation capabilities of GroundingDINO and SAM-2, but this performance heavily depends on predefined target categories and training data distribution. When adapting to other crop or disease types, the front-end segmentation model must be retrained or fine-tuned, preventing rapid deployment. In contrast, the proposed fine-tuning strategy introduces a feature pyramid fusion module into BLAP. With its learnable parameters, this module extracts disease features adaptively without requiring crop-specific front-end processing modules. The experimental results demonstrate that BLAP achieves a disease recognition accuracy of 92.78% across 33 disease/healthy categories covering 11 crop species included in the main dataset. Furthermore, for wheat diseases that were not included in the original training dataset, BLAP still achieved excellent performance after few-shot fine-tuning, demonstrating its strong capability for rapid cross-crop adaptation. It should be emphasized that few-shot cross-crop adaptation and zero-shot cross-crop generalization are fundamentally different concepts. The contribution of this work lies in providing a cost-effective adaptation strategy, whereby only a small number of field images (approximately 108 annotated images per disease category in this study) are required to rapidly adapt the model to a new crop, without the need for training from scratch or full-parameter fine-tuning. In contrast, true zero-shot cross-crop generalization, which requires the model to recognize diseases of previously unseen crop species without any target-domain adaptation, remains challenging due to the difficulty of aligning semantic representations with visual characteristics across different crop species. Addressing this challenge will be an important direction for our future research. Furthermore, the proposed fine-tuning strategy is not inherently tied to the BLIP-2 architecture. In principle, with only minor adjustments and feature dimension alignment, the PFM and APFM modules can be transferred to other vision-language models that share similar cross-modal interfaces, such as InstructBLIP, LLaVA, and Qwen-VL (Liu et al., 2023). However, the effectiveness of such transfer remains to be verified by future experiments. It is worth noting that the generalization advantage of our framework primarily lies in its low-cost adaptability to new crops, achieved by replacing a small number of target-domain labeled samples for cross-crop transfer. This notion differs from the conventional concept of cross-dataset zero-shot generalization, yet it aligns more closely with the practical constraint of high annotation costs in agricultural production. The adaptive visual prompt fusion module accommodates large or dynamically expanding target concept spaces. When encountering new diseases or crops, its learnable visual prompt vectors continuously absorb updated knowledge, circumventing the representation bottleneck of fixed prompt templates. This dynamic adaptability is crucial for open and ever-changing agricultural production scenarios. All trainable modules in BLAP are activated only during training. At inference time, they can be merged with the base model parameters or executed via non-intrusive hooks.

The core design principle of the BLAP framework is to freeze the entire base model and insert lightweight, task-specific adaptation modules at the cross-modal interface, resulting in a flexible fine-tuning mechanism and strong generalization capability. At the text generation level, the adaptive prompt fusion module dynamically controls the injection strength of visual information through learnable visual prompt vectors and a gating mechanism, enabling the model to adjust its language output adaptively based on the generated content. The resulting diagnostic reports balance technical accuracy with readability, support natural language interaction, and facilitate practical deployment. Experiments on field-collected wheat samples demonstrate the few-shot cross-crop adaptation capability of BLAP under complex field conditions.

Regarding the deployment scenario, although the current inference latency of BLAP (approximately 12 s per image) is not suitable for applications requiring millisecond-level real-time responses, it is acceptable for practical agricultural workflows. In typical field applications, farmers and plant protection personnel usually follow a “capture-upload-diagnose” workflow, in which images are captured in the field, uploaded to a cloud or local server, and the diagnostic results are returned after a short waiting period. Therefore, the current inference latency is considered acceptable for such non-real-time scenarios. Furthermore, all trainable modules can be merged into the backbone model after training or executed through lightweight hook mechanisms during inference, facilitating deployment on cloud platforms or edge servers without requiring dedicated high-performance computing hardware.

### 4.2 Limitations and future work

Despite the promising results achieved by BLAP, several limitations remain, which also point to directions for future research.

First, many current large-scale open datasets, despite being standard benchmarks, still fail to fully cover the diverse real-world scenarios encountered in the field. Although approximately 45% of the images in our dataset are collected in the field, these images remain predominantly close-up views of individual leaves with relatively clean backgrounds. The remaining images are captured under controlled laboratory lighting and uniform backgrounds. Overall, the dataset largely sidesteps the practical difficulties posed by varying illumination, occlusions, complex backgrounds, and differences in crop developmental stages in real agricultural environments. This limitation stems from the public data sources used in this work, and we acknowledge it candidly. Future work will focus on collecting and constructing more representative datasets that better reflect the diversity of field conditions, and on exploring domain adaptation techniques to further enhance model robustness.

Second, although the current multi-scale pyramid fusion module incorporates learnable scale weights, these weights are entirely driven by the training data without any explicit prior constraints derived from disease visual characteristics. For diseases with substantially different scale properties, such as spot-type and necrotic-type symptoms, the model should ideally bias its attention toward different scale features automatically. However, under few-shot conditions, purely data-driven weights are prone to settling into suboptimal solutions due to sample bias, thereby increasing the misclassification rate for visually similar diseases. Future work will consider incorporating a scale prior regularization term into the loss function or initializing the fusion weights with dynamic biases conditioned on disease morphological subcategories, so as to reduce the hypothesis space and enhance fine-grained discriminative capability. In the existing adaptive visual prompt fusion module, although the gating mechanism achieves preliminary visual-textual interaction through cross-attention, the current concatenation-plus-MLP design for gate value computation does not fully exploit high-order cross-modal semantic correlations. A promising direction is to introduce a lightweight multi-head attention layer prior to the gating module, enabling the model to more precisely capture the varying demands for visual semantics at different generation positions before deciding the injection strength, thereby further improving the quality of symptom descriptions and treatment recommendations.

Third, the current framework does not explicitly assess disease severity, as the benchmark datasets provide only disease category labels without severity annotations. In future work, we plan to extend the proposed pyramid feature fusion module to estimate lesion severity from multi-scale pathological image features. Specifically, we will leverage lesion regions extracted from different feature levels to compute quantitative indicators, including lesion area ratio, lesion density, and spatial distribution. These indicators will then be projected into learnable severity embedding vectors and fed as additional prompt tokens into the adaptive visual prompt fusion module. In this way, the language model will be able to simultaneously infer both the disease category and the severity level, such as mild, moderate, or severe, while generating corresponding treatment recommendations (He et al., 2025).

Fourth, another limitation is that the generalization of BLAP to diseases with insufficient image and textual information is not yet fully verified. Given that climate change and global trade accelerate the spread and evolution of plant diseases, it is of practical significance to develop models that can reason about emerging diseases and provide preliminary control recommendations. Future work may adopt soft-target contrastive learning integrated with category semantic embeddings, so that visual prompt vectors can infer the characteristics of novel diseases from textual descriptions (Snell et al., 2017). Furthermore, the current framework has not been systematically evaluated for out-of-distribution generalization on public agricultural datasets such as PlantDoc and PlantWild, nor has it been tested with a leave-one-crop-out protocol to quantify cross-crop transferability. These omissions stem primarily from label system differences and inconsistent annotation standards across datasets, which complicate direct attribution of results to model innovations. In addition, the leave-one-crop-out protocol would eliminate all training samples of the target crop, which does not fully match the few-shot fine-tuning setting of this work, as opposed to zero-shot inference. Nevertheless, such evaluations are essential for a comprehensive assessment of model robustness in real-world open scenarios. Subsequent work will design appropriate cross-dataset evaluation protocols and conduct systematic cross-crop generalization experiments to tackle these challenges.

Fifth, the current model relies solely on RGB imagery. Integrating various spectral imaging modalities, environmental data, and unstructured expert knowledge could significantly enhance the model’s decision-making capability. Huang et al. (2025) constructed the FruitMMBench multimodal benchmark and found that mainstream VLMs exhibit error rates ranging from 45% to 75% on fine-grained tasks such as ripeness estimation, underscoring the necessity of domain-specific VLM adaptation. Similarly, Akdoğan et al. (2025) improve YOLO performance with wavelet and histogram preprocessing. Going forward, it will be necessary to design multimodal adapter modules that extend the framework to ingest heterogeneous inputs, and to enrich the system’s perceptual capabilities, ultimately building a more comprehensive and robust crop health perception system.

A sixth limitation concerns the evaluation of generation quality. The current automatic metrics, including BLEU-4, ROUGE-L, and symptom recall, quantify text quality from the perspectives of n-gram order matching, content coverage, and symptom keyword capture, yet they have inherent shortcomings. Both BLEU-4 and ROUGE-L depend on n-gram statistics against reference answers and thus cannot fully assess the semantic plausibility or practical actionability of the generated treatment recommendations. Symptom recall reflects only the coverage of symptom-related keywords and provides no evaluation of the accuracy or completeness of the control advice. In future work, we plan to design an expert evaluation protocol, inviting multiple plant protection specialists to conduct blind reviews and score the generated texts across multiple dimensions, including symptom accuracy, reasonableness of control recommendations, and actionability. Inter-rater reliability, measured by Fleiss’ kappa, will be computed to ensure consistency, thereby providing a more comprehensive assessment of the practical value of our model in real-world agricultural production.

## 5 Conclusion

To address the limitations of general-purpose vision-language models in few-shot crop disease diagnosis, we proposed BLAP, a lightweight adaptive multi-scale visual prompt tuning framework built upon BLIP-2. By introducing a pyramid feature fusion module (PFM) and an adaptive visual prompt fusion module (APFM) between the Q-Former and the large language model, together with LoRA-based parameter-efficient fine-tuning, BLAP effectively enhances multi-scale visual representation and vision-language interaction while keeping the backbone model completely frozen.

Experimental results demonstrate three key advantages of the proposed framework. First, the trainable parameters account for only 0.11% of the full BLIP-2 model, enabling highly parameter-efficient fine-tuning with low computational cost. Second, BLAP improves crop disease recognition accuracy by 21.67% over the BLIP-2+LoRA baseline, achieving an accuracy of 92.78%, while simultaneously generating high-quality diagnostic reports containing symptom descriptions and treatment recommendations. Third, the average inference latency increases by only 2.15%, indicating that the substantial performance improvement is achieved with almost no additional deployment overhead.

Overall, BLAP effectively balances recognition accuracy, language generation quality, parameter efficiency, and inference speed under resource-constrained conditions. The proposed framework provides a practical and lightweight solution for intelligent crop disease diagnosis and offers a general parameter-efficient fine-tuning strategy that can be readily extended to other vision-language models for agricultural applications.

## Data Availability

The original contributions presented in the study are included in the article/supplementary material, further inquiries can be directed to the corresponding author.

## References

[B1] Akdoğan C. Özer T. Oğuz Y. (2025). PP-YOLO: Deep learning based detection model to detect apple and cherry trees in orchard based on histogram and wavelet preprocessing techniques. Comput. Electron. Agric. 232, 110052. doi: 10.1016/j.compag.2025.110052 38826717

[B2] Arshad M. A. Jubery T. Z. Roy T. Nassiri R. Singh A. K. Singh A. . (2024). AgEval: a benchmark for zero-shot and few-shot plant stress phenotyping with multimodal LLMs. arXiv [Preprint]. doi: 10.48550/arXiv.2407.19617

[B3] Chen S. . (2022). Adaptformer: Adapting vision transformers for scalable visual recognition. Adv. Neural Inf. Process. Syst. 35, 16664–16678. doi: 10.52202/068431-1212

[B4] Dai W. . (2023). Instructblip: Towards general-purpose vision-language models with instruction tuning. Adv. Neural Inf. Process. Syst. 36, 49250–49267. doi: 10.52202/075280-2142

[B5] Geetharamani G. Pandian A. (2019). Identification of plant leaf diseases using a nine-layer deep convolutional neural network. Comput. Electr. Eng. 76, 323–338. doi: 10.1016/j.compeleceng.2019.04.011 38826717

[B6] Hassan S. M. Maji A. K. Jasiński M. Leonowicz Z. Jasińska E. (2021). Identification of plant-leaf diseases using CNN and transfer-learning approach. Electronics 10, 1388. doi: 10.3390/electronics10121388 30654563

[B7] He W. Wu F. Snyder L. U. Cheng J. Wilcox E. Xiang L. (2025). Improved two-stage deep learning algorithm and lightweight YOLOv5n for classifying cottonseed damage. Comput. Electron. Agric. 232, 110042. doi: 10.1016/j.compag.2025.110042 38826717

[B8] Houlsby N. Giurgiu A. Jastrzebski S. Morrone B. De Laroussilhe Q. Gesmundo A. . (2019). Parameter-efficient transfer learning for NLP. In: Proceedings of the 36th International Conference on Machine Learning. (Long Beach, CA, USA: PMLR); 2790–2799.

[B9] Hu E. J. Shen Y. Wallis P. Allen-Zhu Z. Li Y. Wang S. . (2022). LoRA: low-rank adaptation of large language models. In: Proceedings of the International Conference on Learning Representations (ICLR).

[B10] Huang G. Xu Z. Liu L. Chen J. He Z. Liu F. (2025). Evaluating and deploying large vision-language models for fruit quality assessment in smart agriculture systems. Comput. Electron. Agric. 238, 110806. doi: 10.1016/j.compag.2025.110806 38826717

[B11] Huang J. Ye J. Hu H. Yang J. Lan W. Zhang Y. (2026). Low-rank adaptation method for fine-tuning plant disease recognition models. Smart Agric. 8 (1), 40–51. doi: 10.12133/j.smartag.SA202504003

[B12] Hughes D. P. Salathé M. (2015). An open access repository of images on plant health to enable the development of mobile disease diagnostics. arXiv [Preprint]. doi: 10.48550/arXiv.1511.08060. Available online at: https://arxiv.org/abs/1511.08060 (Accessed August 12, 2026).

[B13] Huo Z. Yang X. Sun X. Zhang B. Liu D. (2026). EBGC-RTDETR: a lightweight model for eggplant disease detection with enhanced RT-DETR architecture. Smart Agric. Technol. 13, 101761. doi: 10.1016/j.atech.2025.101761

[B14] Jain A. K. (2026). 15 crop and 45 disease and healthy dataset. Mendeley Data. doi: 10.17632/8fr7grr73p.1

[B15] Jia M. Tang L. Chen B.-C. Cardie C. Belongie S. Hariharan B. . (2022). Visual prompt tuning. In: Computer Vision – ECCV 2022. (Cham, Switzerland: Springer); 709–727.

[B16] Jiang X. . (2025). PlantCaFo: An efficient few-shot plant disease recognition method based on foundation models. Plant Phenomics 7, 100024. doi: 10.1016/j.plaphe.2025.100024 41415957 PMC12709961

[B17] Kamilaris A. Prenafeta-Boldú F. X. (2018). Deep learning in agriculture: a survey. Comput. Electron. Agric. 147, 70–90. doi: 10.1016/j.compag.2018.02.016 38826717

[B18] Kaur M. Singh R. Alirezaee S. Hussain I. (2025). Visual-language transformer-based tomato leaf disease detection for portable greenhouse monitoring device. Plant Methods 21, 139. doi: 10.1186/s13007-025-01456-8 41153009 PMC12560288

[B19] Krishna G. Gopal G. (2019). Data for: identification of plant leaf diseases using a 9-layer deep convolutional neural network. Mendeley Data. doi: 10.17632/tywbtsjrjv.1

[B20] Li J. Feng Q. Yang J. Zhang J. Yang S. (2025). Few-shot crop disease recognition using sequence-weighted ensemble model-agnostic meta-learning. Front. Plant Sci. 16, 1615873. doi: 10.3389/fpls.2025.1615873 40842515 PMC12364850

[B21] Li J. Li D. Savarese S. Hoi S. (2023). BLIP-2: bootstrapping language-image pre-training with frozen image encoders and large language models. In: Proceedings of the 40th International Conference on Machine Learning. (Honolulu, Hawaii: PMLR); 19730–19742.

[B22] Li X. L. Liang P. (2021). Prefix-tuning: optimizing continuous prompts for generation. In: Proceedings of the 59th Annual Meeting of the Association for Computational Linguistics and the 11th International Joint Conference on Natural Language Processing (Volume 1: Long Papers). (Online: Association for Computational Linguistics); 4582–4597. doi: 10.18653/v1/2021.acl-long.353

[B23] Liu H. Li C. Wu Q. Lee Y. J. (2023). Visual instruction tuning. Adv. Neural Inf. Process. Syst. 36, 34892–34916. doi: 10.52202/075280-1516

[B24] Liu J. Xing J. Zhou G. Wang J. Sun L. Chen X. (2025). Transfer large models to crop pest recognition—a cross-modal unified framework for parameters efficient fine-tuning. Comput. Electron. Agric. 237, 110661. doi: 10.1016/j.compag.2025.110661 38826717

[B25] Mangrulkar S. Gugger S. Debut L. Belkada Y. Paul S. Bossan B. (2022). PEFT: state-of-the-art parameter-efficient fine-tuning methods. Available online at: https://github.com/huggingface/peft.

[B26] Mohanty S. P. Hughes D. P. Salathé M. (2016). Using deep learning for image-based plant disease detection. Front. Plant Sci. 7, 1419. doi: 10.3389/fpls.2016.01419 27713752 PMC5032846

[B27] Paszke A. Gross S. Massa F. Lerer A. Bradbury J. Chanan G. . (2019). PyTorch: an imperative style, high-performance deep learning library. In: Advances in Neural Information Processing Systems 32 (NeurIPS 2019); 8024–8035.

[B28] Porto J. V. A. Dorsa A. C. Weber V. A. . (2023). Usage of few-shot learning and meta-learning in agriculture: a literature review. Smart Agric. Technol. 5, 100307. doi: 10.1016/j.atech.2023.100307 38826717

[B29] Quoc K. N. Thu L. L. T. Quach L.-D. (2026). A vision-language foundation model for leaf disease identification. Expert Syst. Appl. 299, 130084. doi: 10.1016/j.eswa.2025.130084

[B30] Savary S. Willocquet L. Pethybridge S. J. Esker P. McRoberts N. Nelson A. (2019). The global burden of pathogens and pests on major food crops. Nat. Ecol. Evol. 3, 430–439. doi: 10.1038/s41559-018-0793-y 30718852

[B31] Shi D. Liang L. Chen X. Yang X. Li M. Diao M. (2025). DTM-Unet: A deep learning framework incorporating DenseNet and Transformer for precise identification and quantitative segmentation of cucumber leaf disease. Smart Agric. Technol., 101659. doi: 10.1016/j.atech.2025.101659 38826717

[B32] Shinde S. Attar V. (2024). MH-SoyaHealthVision: an Indian UAV and leaf image dataset for integrated crop health assessment. Mendeley Data. doi: 10.17632/hkbgh5s3b7.1 PMC12175242 40534711

[B33] Snell J. Swersky K. Zemel R. (2017). Prototypical networks for few-shot learning. In: Advances in Neural Information Processing Systems 30 (NeurIPS 2017); 4077–4087.

[B34] Strange R. N. Scott P. R. (2005). Plant disease: a threat to global food security. Annu. Rev. Phytopathol. 43, 83–116. doi: 10.1146/annurev.phyto.43.113004.133839 16078878

[B35] Sultana N. Shorif S. B. Akter M. Uddin M. S. (2022). Cucumber disease recognition dataset. Mendeley Data. doi: 10.17632/y6d3z6f8z9.1 PMC10338290 37456112

[B36] Sun J. Cao W. Fu X. . (2024). Few-shot learning for plant disease recognition: a review. Agron. J. 116, 1204–1216. doi: 10.1002/agj2.21285 41531421

[B37] Sun Q. Fang Y. Wu L. Wang X. Cao Y. (2023). EVA-CLIP: improved training techniques for CLIP at scale. arXiv [Preprint]. doi: 10.48550/arXiv.2303.15389

[B38] Venkatraman S. Kumar S. P. Pandiyaraju V. Abeshek A. Aravintakshan S. (2026). SPROUT: symptom-centric prototypical representation optimization and uncertainty-aware tuning for few-shot precision agriculture. Neurocomputing 667, 132137. doi: 10.1016/j.neucom.2025.132137

[B39] Yan J. Dong B. Guan X. Zheng W. Zhang T. Wang R. (2026). Adaptively fine-tuning and ensembling vision-language model for few-shot image classification. IEEE Trans. Multimedia, 1–14. doi: 10.1109/TMM.2026.3668670

[B40] Yan C. . (2025). CDIP-ChatGLM3: A dual-model approach integrating computer vision and language modeling for crop disease identification and prescription. Comput. Electron. Agric. 236, 110442. doi: 10.1016/j.compag.2025.110442 38826717

[B41] Yang S. Feng Q. Yan W. Zhou W. Yang W. (2025). Recognizing few-shot crop diseases using multimodal-guided visual Transformer. Trans. Chin. Soc. Agric. Eng. 41, 195–203. doi: 10.11975/j.issn.1002-6819.202409189

[B42] Zahweh M. H. Nasrallah H. Shukor M. Faour G. Ghandour A. J. (2023). Empirical study of peft techniques for winter-wheat segmentation. Environ. Sci. Proc. 29, 50. doi: 10.3390/ecrs2023-15833 30654563

[B43] Zhang J. Huang J. Jin S. Lu S. (2024). Vision-language models for vision tasks: a survey. IEEE Trans. Pattern Anal. Mach. Intell. 46, 5625–5644. doi: 10.1109/TPAMI.2024.3369699 38408000

[B44] Zhou Y. Yan H. Ding K. Cai T. Zhang Y. (2024). Few-shot image classification of crop diseases based on vision-language models. Sensors 24, 6109. doi: 10.3390/s24186109 39338855 PMC11435512

